# Hybrid Benzimidazole–Dichloroimidazole Zeolitic Imidazolate Frameworks Based on ZIF-7 and Their Application in Mixed Matrix Membranes for CO_2_/N_2_ Separation

**DOI:** 10.1021/acsami.2c12908

**Published:** 2022-10-04

**Authors:** Qian Jia, Elsa Lasseuguette, Magdalena M. Lozinska, Maria-Chiara Ferrari, Paul A. Wright

**Affiliations:** † EaStCHEM School of Chemistry, University of St Andrews, Purdie Building, North Haugh, St AndrewsKY16 9ST, United Kingdom; ‡ School of Engineering, University of Edinburgh, Robert Stevenson Road, EdinburghEH9 3FB, United Kingdom

**Keywords:** zeolitic imidazolate framework, hybrid ZIFs, Matrimid, PEBAX 1657, mixed matrix membrane, CO_2_/N_2_ selectivity

## Abstract

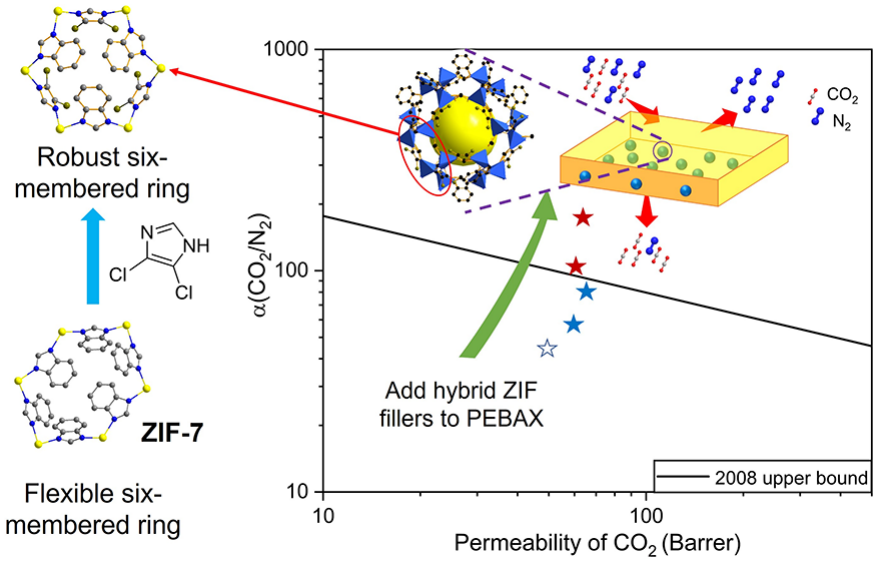

Mixed-linker zeolitic imidazolate frameworks (ZIFs) with the sodalite (**sod**) topology type and based on ZIF-7 have been prepared by direct synthesis from the mixtures of benzimidazole (BzIm) and 4,5-dichloroimidazole (dcIm). Incorporation of dcIm into the ZIF-7 structure gives ZIF-7/COK-17 hybrids with rhombohedral symmetry that do not show the “open-to-closed form” structural transition upon solvent removal exhibited by ZIF-7. They show Type I isotherms for low molecular weight gases and high affinity for CO_2_ even at low partial pressures. Synthesis under mild conditions gives ZIF nanoparticles (250–400 nm) suitable for incorporation into mixed matrix membranes (MMMs): these were prepared with both glassy (Matrimid) and rubbery (PEBAX 1657) polymers. Permeation tests at 298 K and 1.2 bar reveal that the incorporation of Zn(BzIm_0.55_dcIm_0.45_)_2_ nanoparticles at up to ca. 12 wt % gives defect-free membranes with enhanced CO_2_ permeability in both polymer matrices, with retention of selectivity (Matrimid) or with an enhancement in selectivity that is most pronounced for the smaller nanoparticles (PEBAX). The membrane with the best performance exhibits a selectivity of ca. 200 for CO_2_/N_2_ (a 4-fold increase compared to the pure polymer) and a CO_2_ permeability of 64 Barrer. At the relatively low loadings investigated, the MMMs’ performance obeys the Maxwell model, and the intrinsic property of diffusivity of the ZIFs can be extracted as a result.

## Introduction

Anthropogenic CO_2_ emissions to the atmosphere have caused a rapid rise in levels to values (425 ppm) that are expected to cause dangerous extremes in climate change and sea level rise.^1^ To mitigate global CO_2_ emission, carbon capture and storage (CCS) has been proposed and implemented in current carbon-intensive industrial sectors.^2^ Several approaches are being developed for CO_2_ capture, including absorption by solutions of amines or alcohols, porous solid adsorption, and membrane separation.^3^ Among these, membrane-based gas separation possesses the significant advantages of small footprints and considerable energy saving.

Since Monsanto commercialized the Prism membrane for hydrogen separation in the 1980s, there has been strong growth in the use of polymeric membranes for industrial gas separation.^4^ However, most polymeric membranes suffer from a trade-off between gas permeability and selectivity that can be represented by the Robeson upper bound.^5^ Another major concern for this type of membrane is its tendency to show aging, leading to plasticization and a decrease in performance.^6^ The development of mixed matrix membranes (MMMs) by embedding porous filler materials into polymers represents one promising route for solving these issues and improving membrane-based separation.^6^

Microporous metal–organic frameworks (MOFs) have great potential as fillers for MMMs because they possess attributes of high surface areas and well-defined pore sizes, and their surfaces, internal and external, can be easily functionalized and tailored to application. Furthermore, their organic component gives enhanced compatibility with the polymeric membranes compared to that of purely inorganic porous fillers such as zeolites.^2^ Zeolitic imidazolate frameworks (ZIFs) make up a subclass of metal–organic frameworks built from transition metal ions (such as Co^2+^ and Zn^2+^) linked via imidazolate groups into tetrahedrally coordinated frameworks with zeolite-like topologies. The wide variety of available imidazolate linkers enables their porosity and surface chemistry to be tuned. ZIFs have been well studied as fillers for MMMs because, in addition to the features listed above, they show good chemical stability and can readily be prepared in the nanoparticle form (<0.5 μm) suitable for homogeneous dispersion in thin polymer membranes.

ZIFs with the **sod** (sodalite) topology, based on the framework adopted by aluminosilicate sodalite, have attracted the most attention for application in mixed matrix membranes^7−9^ because of their three-dimensionally connected pore volume, which is accessible via six-membered ring windows (six Zn cations and six imidazolate linkers). ZIF-8 (Zn(MeIm)_2_, MeIm = 2-methylimidazolate) and ZIF-94 (Zn(AmeIm)_2_, AmeIm = 4-methyl-5-imidazolecarboxaldehyde), for example, both have the **sod** topology and crystallize with cubic symmetry and have successfully been introduced into MMMs.

ZIF-7 (Zn(BzIm)_2_, BzIm = benzimidazolate), like ZIF-8 and ZIF-94, possesses the **sod** topology, but due to interactions between the bulky BzIm linkers, it does not adopt cubic symmetry but rather exhibits different configurations, depending on the temperature or the presence or absence of guest molecules. These different structures are achieved via different modes of tilting of the imidazolate linkers between the Zn^2+^ cationic nodes. The two most important configurations are wide-pore ZIF-7-I, which has rhombohedral symmetry and a crystallographically-determined pore size of 3 Å, and narrow-pore ZIF-7-II, which is distorted to triclinic symmetry with a smaller pore entrance.^10,11^ The wide-pore ZIF-7-I structure is adopted when in the as-prepared state, containing residual solvent, but when the solvent is removed, the structure converts to the “closed” ZIF-7-II. The phase transition can be reversed by the adsorption of molecules,^12,13^ such as CO_2_, and the two forms are readily distinguished by their X-ray powder diffraction patterns.

ZIF-7 has attractive material properties for use as a filler in MMMs for CO_2_ separation: it shows high CO_2_ uptake and heat of adsorption at a pressure of 1 bar and above, and it can readily be prepared as nanoparticles that can be homogeneously dispersed in polymers. However, the narrow-pore structure that exists when it is activated has windows too small to allow CO_2_ (kinetic radius 3.3 Å) to diffuse through; therefore, it would not be useful in this state, and a phase transition is required to allow significant CO_2_ uptake at room temperature, which only occurs at elevated CO_2_ pressure.^14−16^ Recent studies suggest that this step in adsorption is a result of dynamic effects of the transfer of CO_2_ molecules between pores of different sizes and related changes in the unit cell geometry, at least at a low temperature (195 K).^16^ The transition could give rise to high local strains in the membrane if the interfacial contact between ZIF and the polymer is to be retained, and the form that is thermodynamically stable could vary across a membrane, leading to difficulties in operation.^17−21^ There is some evidence that inclusion of ZIF-7 into MMMs can lock the structure in the open form when polymers with rigid chains are used,^8^ but this may not generally be the case,^8,22^ and therefore, we chose to develop an alternative approach of modifying the structural chemistry of the ZIF itself.

An alternative strategy to retain the attractive properties of ZIF-7, while avoiding the possibility of the phase transition, is by partial or complete linker exchange of BzIm with functionalized BzIm, for example, with −OH or −NH_2_ on the 2-position of the imidazole ring.^23,24^ In this way, 70% replacement of BzIm with 2-amino-BzIm gave ZIF-7 with good properties for CO_2_/CH_4_ separation when incorporated within a cross-linked polyethylene oxide polymer.^24^ Related to this, partial replacement of BzIm with benzotriazolate via postsynthetic modification gave ZIF-7 with a Type I isotherm for CO_2_, which upon incorporation within poly(ether imide) (PEI) displayed some improvement in H_2_/CO_2_ selectivity compared to pure ZIF-7-based MMMs.^25^

However, this approach to improve materials is limited by the availability of different functionalized benzimidazoles. Another synthetic approach is to broaden the linker choice to non-BzIm linkers that are also known to give the rhombohedrally distorted **sod** structure exhibited by ZIF-7. The general approach of mixing linkers with different imidazole “cores” to give hybrid ZIFs is more practicable than postsynthetic modification^26−30^ and has previously been used successfully to prepare ZIF-7/ZIF-8 hybrids (BzIm/MeIm) in the cubic **sod** form with tunable aperture size, for example.^31,32^ However, that study showed that there is a threshold of 35% for BzIm in the cubic **sod** structure due to the difference in the structure (ZIF-7, *R*3̅ space group compared to ZIF-8, *I*4̅3*m*), while in *R*3̅ ZIF-7, BzIm is the dominant ligand (>90% inclusion). Furthermore, attempts to prepare hybrid ZIF-7/ZIF-90 (BzIm/aldIm, aldIm = 2-imidazolecarboxaldehyde) have been made, but the product ZIFs showed complex, solvent-dependent phase crystallization, with five different phases being observed as the linker ratio was changed.^33^ These results indicate that mixing of imidazole linkers that separately give ZIFs with different crystal structures does not usually give extended solid solutions with gradual changes in properties simply by adjusting the linker ratio.

Here, we report the preparation of hybrid ZIFs based on ZIF-7 by introducing 4,5-dichloroimidazole (dcIm) as a second linker. This dcIm has been reported as the sole linker in **rho**-type ZIF-71 and **lsc**-type ZIF-72.^34^ Previous study has observed that the corresponding **sod**-type Zn(dcIm)_2_ can be achieved by modulated synthesis directing toward either the rhombohedral (isostructural to ZIF-7) or metrically cubic form. Notably, the metrically cubic form is a disordered material where the structure locally has the geometry of the rhombohedral form, with its trigonal axis aligned with the diagonal triads of the overall body-centered cubic structure.^35,36^ The rhombohedral structure was found to be the most stable by computational simulations.^37^ Recently, Wee et al. also showed that via a careful choice of solvent, dcIm can directly give a zinc ZIF with the rhombohedrally distorted **sod** framework with a similar unit cell size to ZIF-7, which they named COK-17.^38^ Furthermore, it showed no transition to a narrow-pore form when activated and gave Type I isotherms for CO_2_ with considerably higher heats of adsorption than ZIF-8.

In this study, in the light of the structural similarity and excellent reported adsorption properties of the end-member ZIFs, ZIF-7 and COK-17, over different ranges of partial pressure of CO_2_, one-pot syntheses using rapid mixing^39^ were adjusted to prepare hybrid ZIF-7/COK-17 materials with different linker ratios and particle sizes, to the best of our knowledge for the first time. Subsequent characterization shows them to possess the desired material advantages over each of the ZIF end-members (no phase transition; suitable particle size and morphology), so these hybrid ZIF nanoparticles were incorporated as fillers into two polymer matrixes, rubbery PEBAX 1657 and glassy Matrimid, with the aim of preparing MMMs with enhanced CO_2_/N_2_ separation properties. These properties were determined for both families of membranes by the measurement of selectivity and permeability under conditions relevant to carbon capture (ca. 1 bar, room temperature).

## Results and Discussion

### Characterization of ZIF Materials

**Figure 1:**
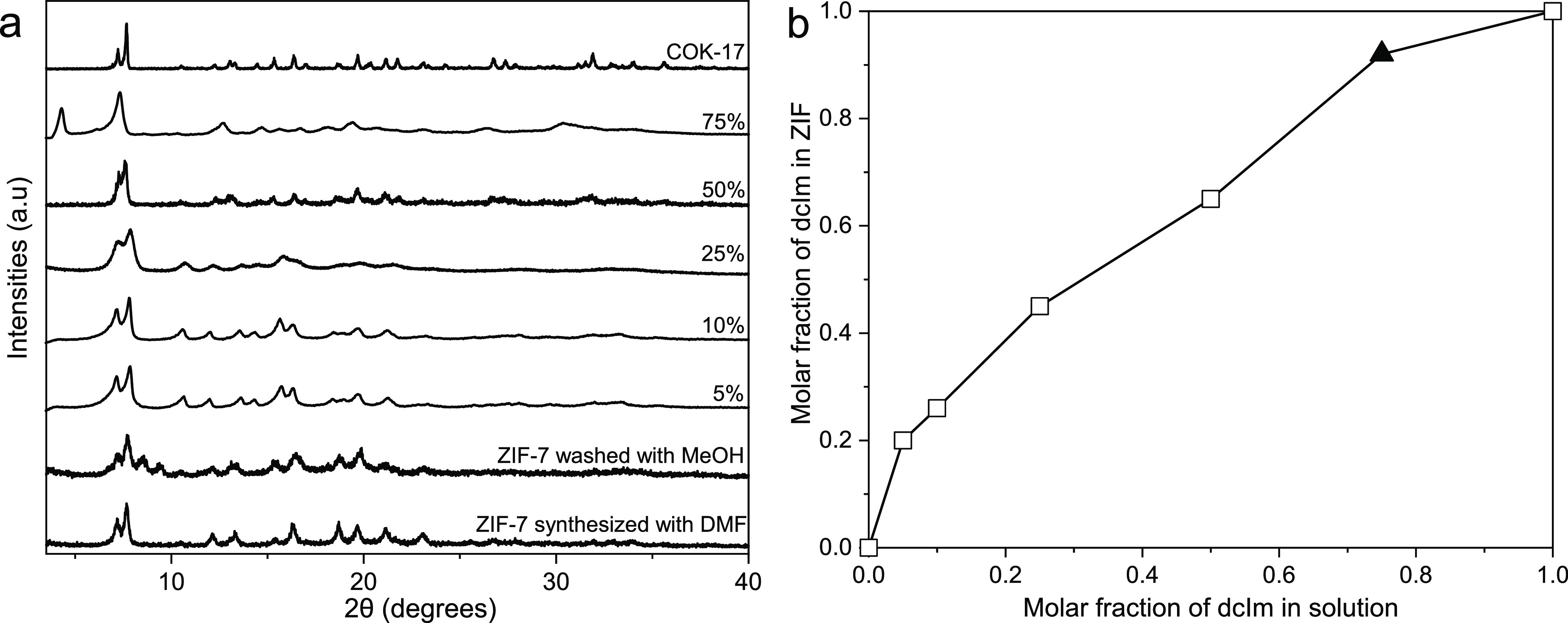
(a) PXRD patterns of hybrid ZIFs with BzIm and dcIm compared to those for ZIF-7-I and COK-17. The percent of dcIm in the syntheses is given. All synthesized ZIFs washed with methanol to remove DMF and allowed to dry. (b) The inclusion of dcIm in hybrid ZIF versus the molar fraction of dcIm in the synthesis solution (dcIm/(dcIm+BzIm)): open squares represent the rhombohedral **sod** structure, and the solid triangle indicates the **rho** structure.

For inclusion in MMMs, a material with good CO_2_ uptake over all partial pressures was required in the nanoparticulate form that could be dispersed in polymer membranes. Syntheses using Zn(NO_3_)_2_ and BzIm or dcIm or mixtures of the two imidazole linkers at room temperature (i.e., 293 K) gave ZIF-7 and a series of hybrid ZIFs. It was not possible to crystallize the Zn(dcIm)_2_ end-member at room temperature, but a sample was prepared at 393 K to enable comparison. Initial studies showed that while the use of methanol as a synthesis solvent favors the crystallization of ZIF BzIm/dcIm hybrids with the **rho** topology (Figure S1), the use of dimethylformamide (DMF) gives the “ZIF-7” rhombohedral **sod** structure up to high concentrations of added dcIm (Figure 1a). It should be noted that ^1^H NMR spectra of dissolved samples (Figure S2; see Materials and Methods for details) enabled quantification of the amount of the dcIm linker in these hybrid ZIFs (Figure 1b). The larger pore size of the **rho** structure results in lower interactions with CO_2_ than those observed with the **sod** framework (a comparison of uptake on **sod** and **rho** hybrid ZIFs with 45 mol % dcIm is given in Figure S3), and these materials were not considered further in this study.

These results show that a rhombohedral **sod** material could be prepared with as high as 65 mol % dcIm being incorporated. In general, dcIm in the resulting hybrid ZIFs is incorporated at a higher level than that present in the synthesis mixture, which implies that dcIm is favored during crystallization into the rhombohedral ZIF structure. In the subsequent discussion, the hybrids are described with the nomenclature ZIF-7/COK-17*_xx_*, where *xx* is the percent of the dichloroimidazole linker in the hybrid ZIF. Compared to hybrid ZIF-7/ZIF-8,^24^ the hybrid ZIF-7/COK-17 has a much wider range of incorporation of the second non-Bzlm linker into the rhombohedral **sod** structure.

**Figure 2:**
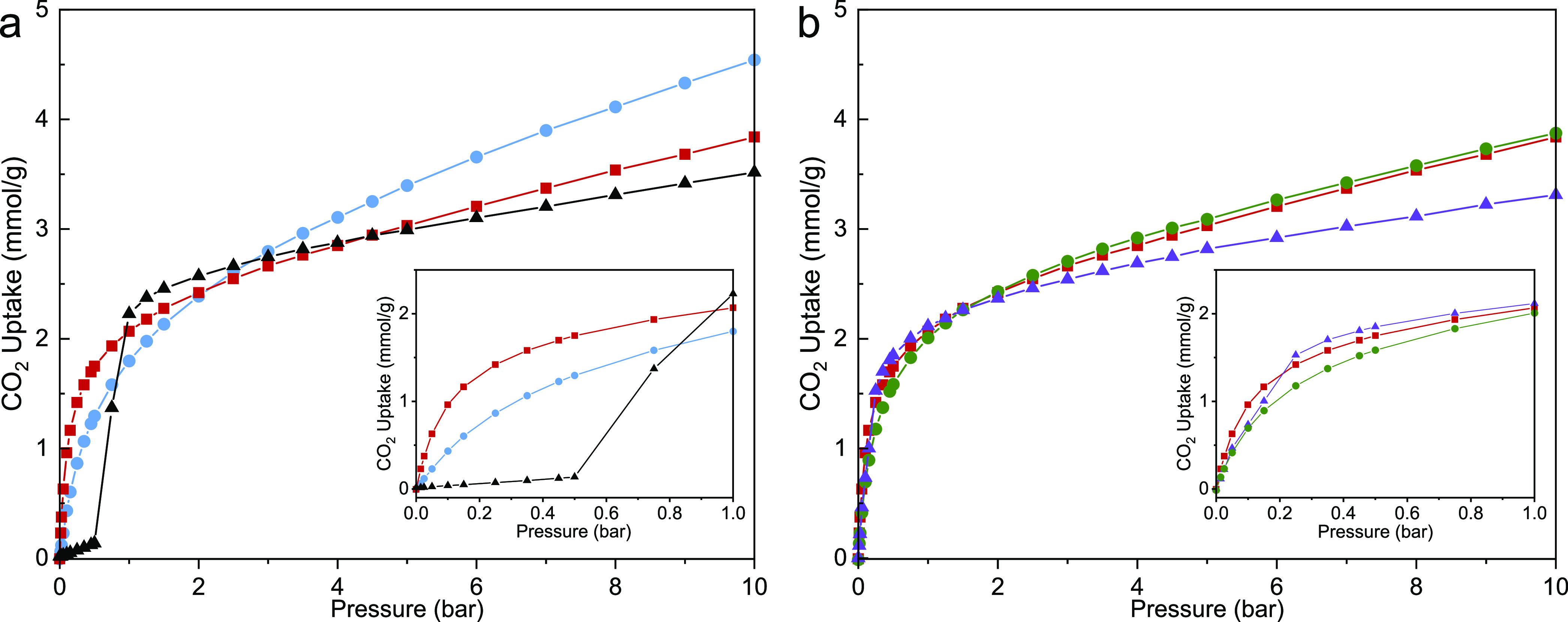
CO_2_ isotherm adsorption at 298 K: (a) hybrid ZIF-7/COK-17_45_ (red squares) compared with ZIF-7 (black triangles) and COK-17 (blue circles); (b) hybrid ZIF-7/COK-17_45_ (in red) compared with hybrid ZIFs with 20% (purple triangles) and 65% (olive circles) dcIm inclusion.

In each member of the series, DMF remaining from the synthesis was removed by washing with methanol and the sample was dried. ZIF-7 undergoes a phase transformation to “closed”-pore ZIF-7-II upon removing MeOH, indicated as additional reflections between 2θ values of 7.5 and 10°, for example, none of the materials with included dcIm show this transition (Figure 1a). CO_2_ adsorption isotherms of these samples (Figure 2a) indicate that ZIF-7 shows its characteristic stepped isotherm and low CO_2_ uptake below ca. 1 bar, and the hybrid ZIF with 20% dcIm also shows some non-Type I behavior at p_CO2_ < 0.25 bar that implies a pore opening from a structure where interactions between the BzIm linkers are important in the activated form (Figure 2b). At higher dcIm levels (45 and 65%), the isotherms are Type I in shape, with higher specific uptakes than ZIF-7 at 10 bar due to the additional pore volume. Of the samples, the CO_2_ behavior of ZIF-7/COK-17_45_ is most promising, taken over the whole range.

**Figure 3:**
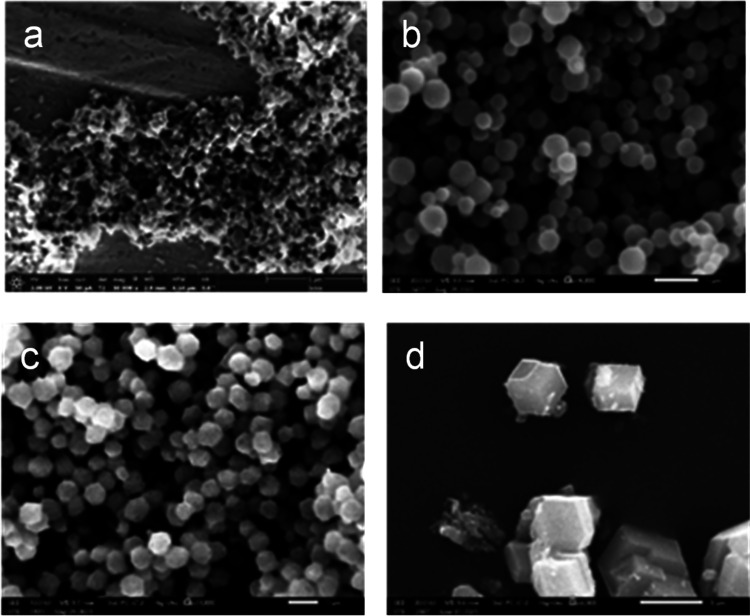
SEM images of (a) ZIF-7, (b) ZIF-7/COK-17_45_, (c) ZIF-7/COK-17_65_, and (d) COK-17.

The SEM images of the prepared ZIF crystallites are given in Figure 3. The crystallite size of ZIF-7 (Figure 3a) is very small (ca. 70 nm), while that of the hybrids is a little larger (Figure 3b,c), but still acceptable for inclusion in mixed matrix membranes (ca. 400 nm).^40^ Notably, the shape of the nanoparticles becomes more clearly faceted and nonspherical as the percentage of dcIm is increased, with morphology approaching that of the much larger, well-faceted crystals of COK-17 (Figure 3d).

Attempts were made to prepare MMMs of some of these materials. The small crystals of ZIF-7 tended to agglomerate when blending with a polymer and do not give a homogeneous dispersion within the polymer matrix (Figure S4a), while crystals of COK-17 are too large for the preparation of MMMs. Among the hybrids, faceted ZIF-7/COK-17_65_ (Figure 3c) gives membranes with pin holes (Figure S4b), but ZIF-7/COK-17_45_ was found to give defect-free membranes, and due to this and its promising CO_2_ adsorption behavior, it was considered further.

**Figure 4:**
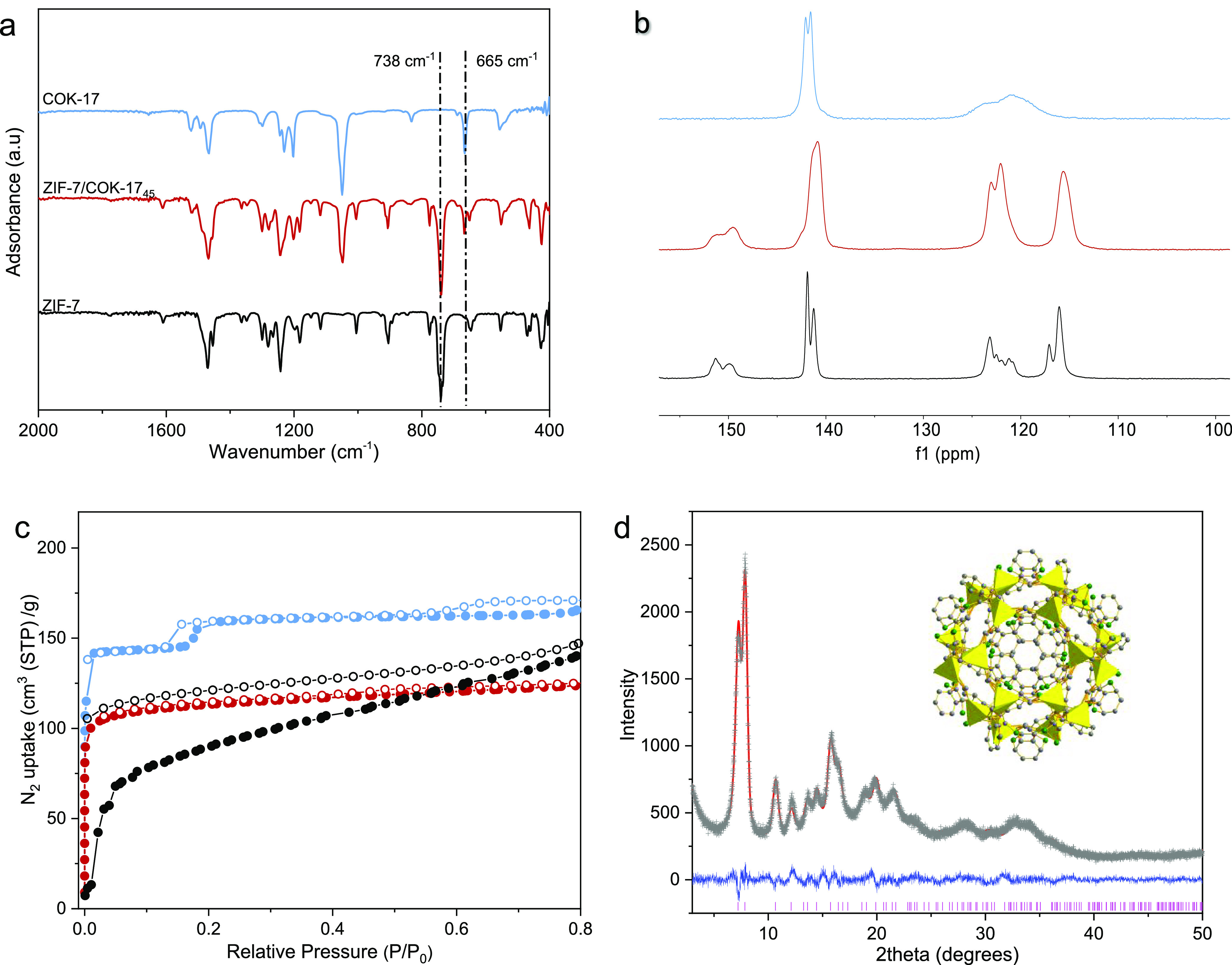
ZIF-7/COK-17_45_ compared with ZIF-7 and COK-17: (a) FTIR spectra; (b) solid-state ^13^C MAS NMR spectra; (c) N_2_ adsorption isotherm at 77 K (ZIF-7 (black), COK-17 (blue), and ZIF-7/COK-17_45_ (red)); and (d) Rietveld refinement of the activated ZIF-7/COK-17_45_ with a *sod* cage inset (calculated data in the red solid line; experimental data in the grey dash line; the difference between calculated and experimental data is in the blue solid line; and Bragg peak markers in pink).

The presence of dcIm in ZIF-7/COK-17_45_ is confirmed by FTIR and SS–NMR (Figure 4a,b). In IR spectra, the distinct peaks at 738 and 665 cm^–1^ are attributed to imidazole in-plane ring bending and C–Cl stretching, respectively, confirming the inclusion of both BzIm and dcIm linkers. In the solid-state NMR spectra, broadening in ^13^C signals from the BzIm linker as dcIm is included indicates the generation of disorder in a single rhombohedral **sod** phase. The thermal stability of ZIF-7/COK-17_45_ was also compared to the end-members ZIF-7 and COK-17 (Figure S5). Three ZIFs experience different weight loss: COK-17 begins to lose significant mass, due to decomposition of the structure, at 688 K, while ZIF-7/COK-17_45_ begins to decompose at 708 K, and ZIF-7 shows the highest decomposition temperature above 773 K. This is related to the lower bond strength of C–Cl in dcIm. A typical Type I N_2_ adsorption isotherm at 77 K is observed in the hybrid ZIF-7/COK-17_45_, while the two end-members exhibit steps with hysteresis at different stages, implying the occurrence of phase transformation between open-pore and closed-pore structures (Figure 4c).^12,38^ Rietveld refinement for ZIF-7/COK-17_45_ in the dehydrated form confirms the disordered presence of dcIm in the framework (Figure 4d) with more details given in Table S1.

**Figure 5:**
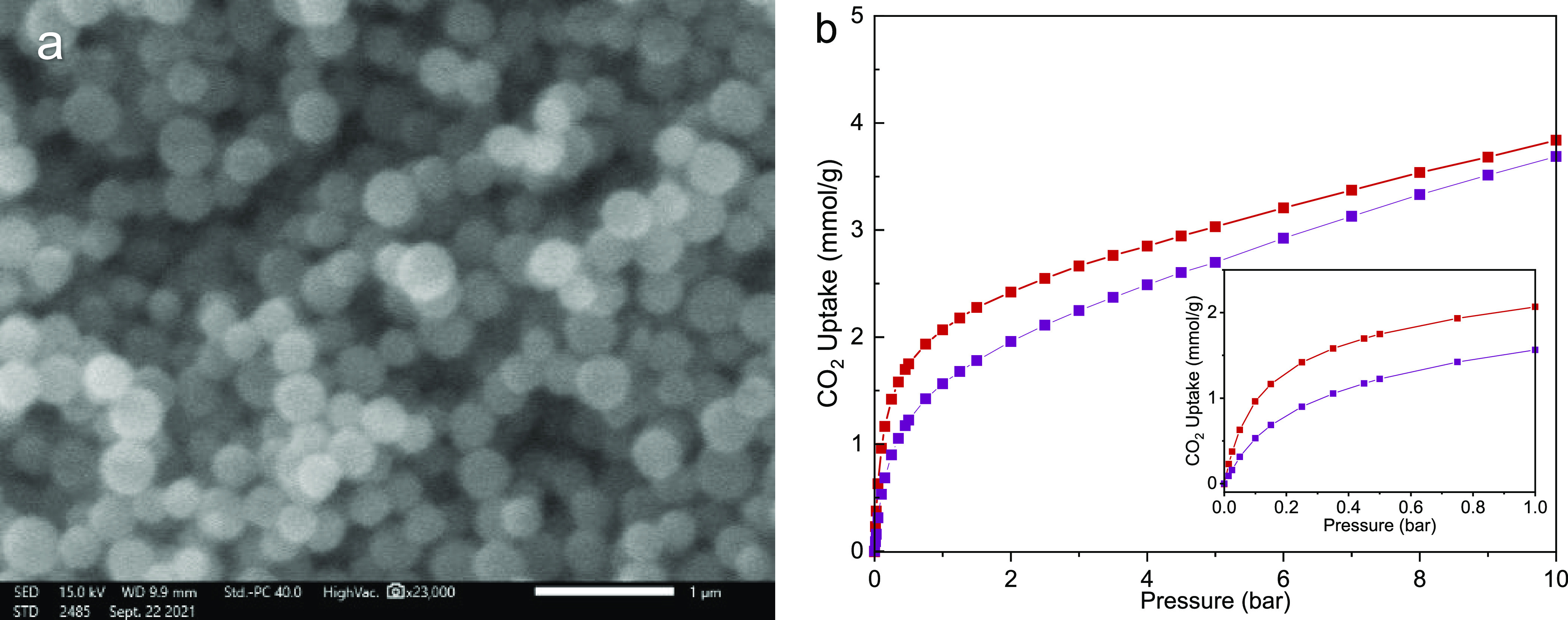
(a) SEM image of ZIF-7/COK-17_45_ (S) and (b) CO_2_ adsorption isotherm at 298 K of ZIF-7/COK-17_45_ (S) (in purple) compared to ZIF-7/COK-17_45_(L) (in red).

Having chosen the 55% BzIm and 45% dcIm composition as optimum for MMMs, the synthesis temperature was modified to reduce the size of the nanoparticles, which can have advantages for use in MMMs.^41^ Lower temperatures have been reported previously to give smaller nanoparticles.^42^ ZIF-7/COK-17_45_ was therefore synthesized at 258 K. As shown in SEM images (Figure 5a), ZIF prepared at 258 K (referred to as ZIF-7/COK-17_45_(S)) has a particle size of around 250 nm compared to 400 nm for that prepared at 293 K (subsequently referred to as ZIF-7/COK-17_45_(L)). The broad peaks in the PXRD pattern (Figure S6) are consistent with a smaller crystal size or local strains, and no Rietveld refinement was attempted. ^1^H NMR of spectra of the dissolved ZIFs indicate very similar linker ratios in the two ZIFs. The CO_2_ uptake of ZIF-7/COK-17_45_(S) was also measured and compared to that of ZIF-7/COK-17_45_(L) (Figure 5b). ZIF with a smaller crystallite size is less crystalline compared to the same composition prepared at a higher temperature, which results in a slightly lower CO_2_ uptake.

In summary, for further studies, the 45% hybrid with 450 nm particles was investigated as a filler because it shows suitable properties of both CO_2_ adsorption and particle morphology and size. The specific CO_2_ uptakes at 298 K at 0.1, 1, and 10 bar are compared favorably with “open” ZIF-7 hybrids reported previously (Table S2).^24^ The linkers are shown to be distributed throughout the particles by the presence of a single phase with broadened diffraction peaks, and this explains why the properties are intermediate between those of the end-members. Additionally, smaller nanoparticles of a hybrid ZIF with a similar composition serve to enable the comparison of the role of filler particle size in determining MMM properties, although the slightly lower CO_2_ adsorption capacity, likely a result of lower crystallinity, should be taken into account.

### Mixed Matrix Membranes

Flat-sheet MMMs of the two hybrid ZIFs in the polymers Matrimid 5218 and PEBAX 1657 have been fabricated by the solution casting method to give membranes at around 30–50 μm in thickness, as described in the Materials and Methods section.

#### Morphology

**Figure 6:**
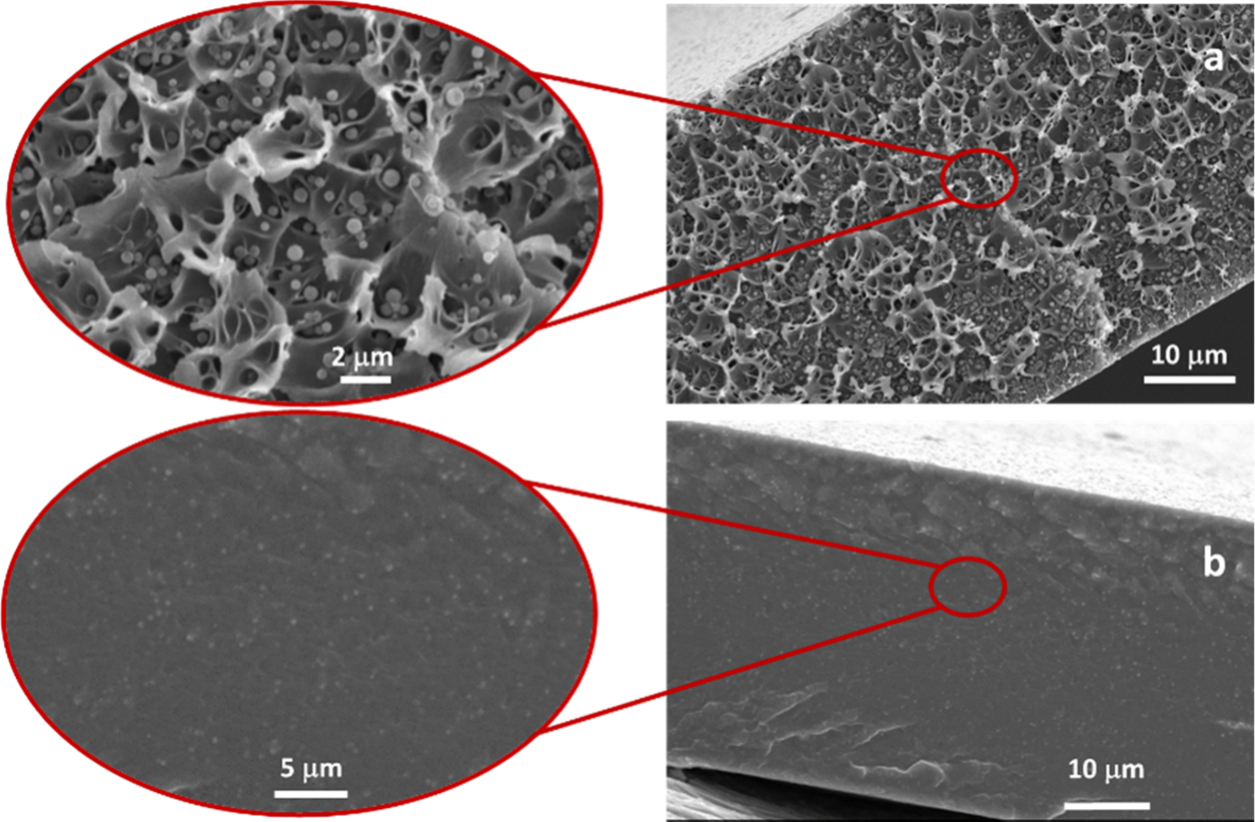
SEM cross-sectional images from Matrimid_ZIF-7/COK-17_45_(L)-8% (a) and PEBAX_ZIF-7/COK-17_45_(S)-5% (b).

For mixed matrix membranes, MOF filler particle size and morphology influence the degree of their agglomeration within the polymer and so are of great significance in membrane performance.^43^ The SEM images shown in Figures 6 and S7–S12 demonstrate good dispersion of the fillers independent of the ZIF and polymers used. Differences in membrane texture can be appreciated when comparing Matrimid and PEBAX membranes due to the different physical states of the two polymers. Matrimid is a glassy polymer and there is more roughness (due to fractures) visible on the cut surface, while rubbery PEBAX appears more uniform. From the surface and cross section, no defects or agglomeration are observed in either case, indicating that MOF particles are uniformly distributed throughout the polymer matrix for both PEBAX and Matrimid regardless of doping concentration and particle size. The SEM images show that the number of filler particles encapsulated within a polymer matrix at a fixed volume fraction increases as particle size decreases. Furthermore, the homogeneity of ZIF filler dispersions within the polymers is confirmed by elemental mapping of Zn and Cl by SEM/EDX (Figure S13). The particle size measured from the SEM images (Table S3) corresponds to that of the as-synthesized particles, indicating that the ultrasonication process does not affect the particle structure. The crystallinity of ZIF fillers is retained after mixing with polymers (Figure S14), although the first two peaks from the ZIF-7 structure are broadened and merge into a single peak, which could be due to the diffusion of polymers into the ZIF pores. It should also be noted that the addition of ZIF fillers can accelerate membrane decomposition upon heating under air: with an increase in ZIF loading, the onset temperature of decomposition is reduced from 473 to 443 K (Figure S15).

#### Gas Permeation

**Figure 7:**
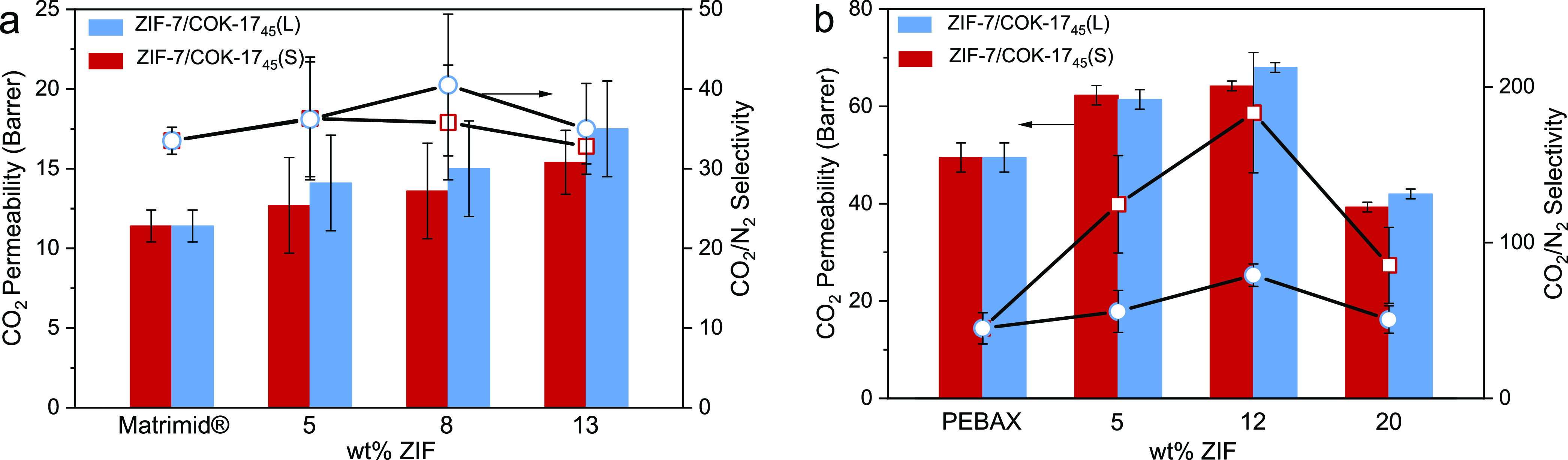
CO_2_ permeability and CO_2_/N_2_ selectivity for (a) Matrimid-based membranes and (b) PEBAX-based membranes versus wt % ZIF (blue: ZIF-7/COK-17_45_(L); red: ZIF-7/COK-17_45_(S)).

The permeation of pure N_2_ and CO_2_ gases through neat polymer membranes and the MMMs was evaluated at 1.2 bar absolute and 298 K. Figure 7a,b shows the gas permeation properties for MMMs based on Matrimid and PEBAX, respectively. The addition of ZIF up to 12 wt % induced an increase in CO_2_ permeability regardless of matrix nature and ZIF particle size. This could be related to an increase in the composite affinity to CO_2_, a disruption of the chain packing of the polymer^6,7^ and an increase in the polymer free volume.^44−46^ At higher filler content in PEBAX, the gas permeability and selectivity decreased; this is most likely related to the formation of filler agglomerates combined with the interaction of polymer chains with the fillers and consequent pore blockage.

To understand this MMM behavior, CO_2_ solubility and diffusivity coefficients have been determined. Table S4 summarizes permeability, diffusivity, and solubility coefficients for the two gases and the two ZIF fillers. As the MMMs are considered dense membranes, the solution-diffusion mechanism can be applied considering the permeability as the product of solubility and diffusivity. Therefore, diffusivity coefficients can be calculated from experimental curves using eq 3 in the Materials and Methods section, while solubility coefficients are obtained from eq 4 in the Materials and Methods. For Matrimid-based MMMs, the change in selectivity is less pronounced and in the order of the measurement error (Figure 7b). Therefore, in this work, we put emphasis on PEBAX-based MMMs.

**Figure 8:**
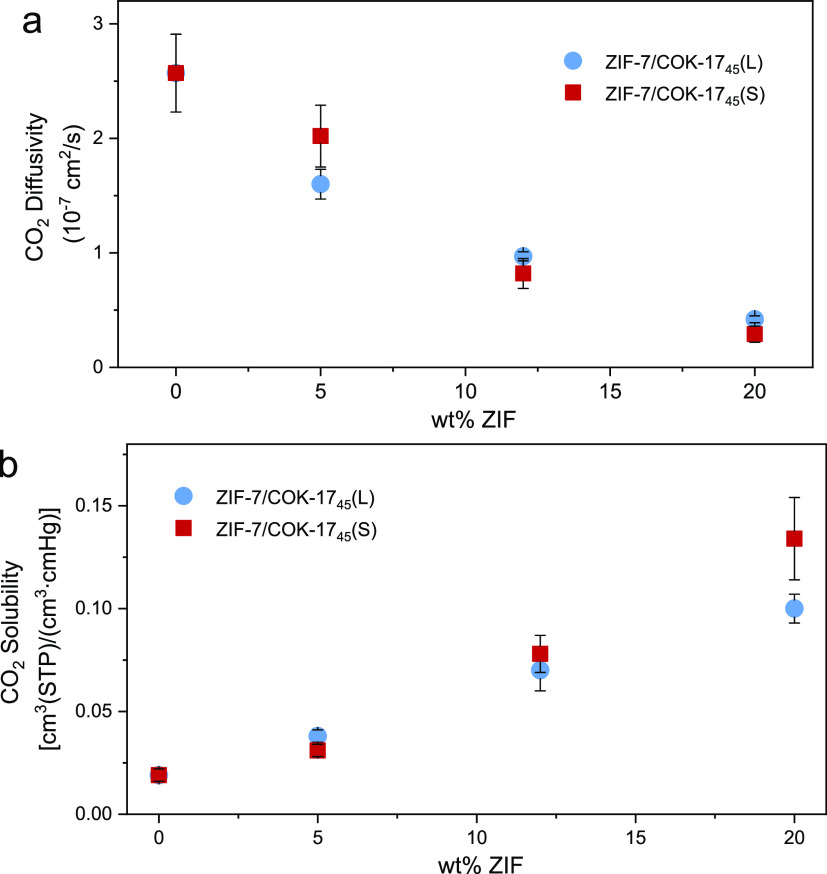
(a) CO_2_ diffusivity and (b) solubility for PEBAX-based membranes as a function of the wt % of ZIF (blue: ZIF-7/COK-17_45_(L); red: ZIF-7/COK-17_45_(S)).

As shown in Figure 8a,b, the addition of ZIF induces a decrease in the diffusivity coefficient and an increase in the solubility coefficient for CO_2_ regardless of the particle size. The decrease in diffusivity can be attributed to some penetration of the flexible PEBAX chains (polyether segments) into the MOF pores, as suggested by the broadening of the PXRD patterns of ZIF in the MMMs.^24,44^ Also, the interaction between the filler and the polymer matrix may disturb the packing and rotational mobility of the polymer chains and thus influence the overall diffusion properties.^6^ The solubility enhancement is explained by the high CO_2_ solubility of ZIF-7/COK-17_45_, which is almost 20 times higher than that of neat PEBAX (Table 1). This improvement contributes to the increase of CO_2_ permeability. At the highest loading (20 wt %), the lower diffusivity is also due to the larger tortuosity introduced by the agglomerates, and it is not fully compensated by the increase in solubility due to the solubility of the filler, leading to lower overall permeability. The CO_2_ solubility data obtained from the permeation measurement for the samples PEBAX_ZIF-7/COK-17_45_(S)-12% and PEBAX_ZIF-7/COK-17_45_(L)-12% were compared to those obtained by the sorption measurement (Figure S16) with very good agreement between the two techniques (Table 1).

**Table 1:** CO_2_ Solubility at 1.2 bar and 298 K

sample	CO_2_ solubility [10^–2^ cm^3^(STP)/(cm^3^·cmHg)]	experiment
ZIF-7/COK-17_45_(L)	42	sorption
ZIF-7/COK-17_45_(S)	34	sorption
PEBAX	1.9	permeation
PEBAX_ZIF-7/COK-17_45_(L)-12%	6.9	permeation
	6.2	sorption
PEBAX_ZIF-7/COK-17_45_(S)-12%	7.8	permeation
	7.1	sorption

For PEBAX-based membranes, the permeability of N_2_ decreased as the filler loading increased (Table S4), which induces an increase in selectivity, as shown in Figure 7b. This might be explained by the molecular sieving effect of hybrid ZIF-7/COK-17_45_ on the larger nitrogen molecule.^8,47^ This increase in selectivity is higher for ZIF-7/COK-17_45_(S), i.e., the smaller particle size. The impact on N_2_ permeability variation is larger for ZIF-7/COK-17_45_(S) than that for ZIF-7/COK-17_45_(L) (Table S4). For instance, with 12 wt % MOF, the N_2_ permeability of PEBAX_ZIF-7/COK-17_45_(S) decreased by 68%, whereas for PEBAX_ZIF-7/COK-17_45_(L), it decreases by only 22% compared to the neat PEBAX. Zheng et al. reported similar behavior with PEBAX mixed with ZIF-8, with larger CO_2_/N_2_ selectivity for the smaller size of ZIF-8.^48^ The authors explained that with a smaller particle size, the external area increases, which contributes to more active sites for CO_2_ capture and large mass transfer resistance for N_2_ due to its larger kinetic diameter. Moreover, the number of fillers incorporated in the MMM at a fixed volume fraction increases as particle size decreases. This induces larger tortuosity and mass transfer resistance within the MMM based on ZIF-7/COK-17_45_(S) and thus lower N_2_ permeability.

These results were compared to other PEBAX-ZIF hybrid membranes reported in the literature (Table 2).^6,8,47−51^ The membranes with ZIF-7/COK-17_45_(S) achieved a significant enhancement in CO_2_/N_2_ selectivity with no detriment to the permeability, while other studies reported an increase in permeability but no gains for the selectivity. This difference can partially be attributed to the preparation of the membrane (PEBAX and MMM). The fabrication conditions (i.e., temperature, ultrasound treatment) have an impact on the separation performances of PEBAX,^51^ which explains the difference in the performance of the pure polymer in the literature. As a consequence, the addition of selective fillers can have a lower relative impact in some cases.

**Table 2:** Performance of Recently Published PEBAX 1657-Based Mixed Matrix Membranes with ZIF Materials Incorporated as Fillers

materials	P_CO2_ [Barrer]	P_CO2_ increment [%]	CO_2_/N_2_ selectivity	selectivity increment [%]	measurement condition	refs
PEBAX	45		60		1.2 bar and 298 K	(6)
ZIF-94[25%]	59	31	53	–13		
PEBAX	70		34		3.75 bar and 308 K	(8)
ZIF-7[8%]	145	107	68	100		
PEBAX	80		50		1 bar and 298 K	(47)
IL@ZIF-8	105	31	84	68		
PEBAX	75		45		1 bar and 293 K	(48)
ZIF-8(NP)[10%]	120	60	52	16		
PEBAX	100		34		2 bar and 297 K(100%RT)	(49)
ZIF-C	387.2	287	47.1	39		
PEBAX	70		50		11 bar and 308 K	(50)
ZIF-67[5%]	162	131	81	62		
PEBAX	49		47		1.2 bar and 298 K	(51)
ZIF-8[10%]	84	71	62	32		
PEBAX	49.5		45		1.2 bar and 298 K	this work
ZIF-7/COK-17_45_(S)[5%]	62.3	26	124.6	180		
ZIF-7/COK-17_45_(S)[12%]	64.2	30	183.4	310		

The Maxwell model is usually used to fit the permeability versus filler fraction performance for relatively low volume fractions of fillers when ideal behavior can be assumed. In this case, it was used to obtain an insight into the intrinsic CO_2_ permeability of the ZIF fillers that could not be measured directly. The gas-transport properties of ZIF-7/COK-17_45_(L) and ZIF-7/COK-17_45_(S) were calculated from the experimental permeation data of PEBAX_ZIF-7/COK-17_45_ at 12 wt % loading using eq 6 of the Materials and Methods section.^52^ The CO_2_ permeabilities of ZIF-7/COK-17_45_(S) and ZIF-7/COK-17_45_(L) are estimated to be 549 and 2905 Barrer, respectively. Furthermore, using the measured CO_2_ sorption data (Table 1), it is possible to retrieve the intrinsic CO_2_ diffusivity of the MOF samples considering that the effective permeability is the product of solubility and diffusivity (eq 4 of the Materials and Methods section). As shown in Table 3, ZIF-7/COK-17_45_(L) presents higher solubility and diffusivity compared to that with smaller particles.

**Table 3:** Intrinsic Properties of ZIF-7/COK-17_45_(L) and ZIF-7/COK-17_45_(S), ZIF-7, and ZIF-7-NH_2_, where the CO_2_ Permeability was Calculated from the Maxwell Eq (6), Solubility was Measured from CO_2_ Adsorption, and Diffusivity was Calculated from Eq (4)

MOF	CO_2_ permeability [Barrer]	CO_2_ solubility [cm^3^(STP)/(cm^3^·cmHg)]	CO_2_ diffusivity [10^–7^ cm^2^/s]	measurement condition
ZIF-7/COK-17_45_(L)	2905	0.42	6.9	1.2 bar and 298 K single gas
ZIF-7/COK-17_45_(S)	549	0.34	1.6	

To assess the validity of application of the Maxwell equation to give the permeability for pure ZIF fillers, the Maxwell model was again applied, in this case, to predict the performance of the MMMs based on Matrimid. As shown in Figure S17, the experimental data match well with the theoretical prediction, confirming the reliability of the pure filler data calculated from MMM data and suggesting close to ideal behavior at the interface of the ZIF and the polymer in the composite.

## Conclusions

The synthesis of hybrid BzIm/dcIm ZIFs with the rhombohedral **sod** structure has been performed, to our knowledge for the first time, by replacement of up to 65% of the benzimidazole linker in the ZIF-7 structure by 4,5-dichloroimidazole (dcIm) via rapid, low-temperature, one-pot synthesis. By lowering the synthesis temperature to subambient, the particle size of these hybrid ZIFs can be reduced to 250 nm.

This family of hybrid ZIFs displays high CO_2_ uptake and desirable nanoparticle size and morphology that enable good dispersion in mixed matrix membranes (MMMs). Furthermore, incorporation of the dcIm linker inhibits adoption of the closed form (seen for pure ZIF-7), which exhibits strongly reduced CO_2_ uptake at low partial pressures.

Hybrid BzIm/dcIm ZIF-7/COK-17_45_ nanoparticles can be incorporated homogeneously and without aggregation into the glassy Matrimid 5218 and the rubbery PEBAX 1657 polymers and prepared as flat, defect-free membranes a few tens of μm in thickness. These have been tested for the permeation of pure N_2_ and CO_2_ at 298 K and 1.2 bar. Increasing their loading up to 12–13 wt % significantly increases the permeability of both membranes (by ca. 40–60%). This was achieved without loss of selectivity for the Matrimid MMMs and with strongly increased CO_2_/N_2_ selectivity for the PEBAX MMMs, particularly by inclusion of the smaller nanoparticles. Possible mechanisms for this increase in selectivity include modification of the PEBAX polymer structure and increased molecular sieving in the smaller nanoparticles. In any case, the observed improvements demonstrate the potential for MMM performance improvement of the modification of structure and morphology by compositional tuning in mixed-linker MOF fillers.

It was also found that at these relatively low filler contents, the Maxwell model gave a good fit to permeability data for MMMs based on the two types of polymers. Where this criterion is met, the approach can yield values for filler diffusivity, intrinsic to the ZIF particles, that could not readily be obtained directly due to the practical difficulties inherent to the preparation of pure defect-free MOF membranes.

## Materials and Methods

### Materials

PEBAX MH1657 (Arkema), Matrimid 5218 (Hunstman), zinc nitrate hexahydrate (Alfa Aesar, 98%), benzimidazole (BzIm, Alfa Aesar, 99%), 4,5-dichloroimidazole (dcIm, Fluka, 98%), N,N-dimethylformamide (Acros Organics, 99%), and methanol (99.9%) were used in the paper.

### Synthesis of ZIFs

ZIF-7 nanoparticles were synthesized by dissolving 2 mmol of Zn(NO_3_)_2_·6H_2_O and 8 mmol of benzimidazole in 50 mL of DMF. After stirring vigorously for 2 h, the product was collected by centrifugation with a speed of 14 500 rpm and then washed with fresh DMF three times. The powder was activated with nitrogen flow for 10 h.

COK-17 particles were synthesized according to the protocol from Wee et al.^38^ First, 2 mmol of Zn(NO_3_)_2_·6H_2_O was dissolved in 100 mL of a mixture of DMF and H_2_O with a ratio of 19:1. Then, 4.3 mmol of 4,5-dichloroimidazole was subsequently added to the solution. After thorough mixing, the synthesis solution was heated at 393 K for 4 days and kept stirring and static for 3 days. The powder was collected by centrifugation at 14 500 rpm and then washed with fresh DMF three times. The powder was activated with nitrogen flow for 10 h.

For the synthesis of hybrid ZIF with 4,5-dichloroimidazole (**sod**), 2 mmol Zn(NO_3_)_2_·6H_2_O was dissolved in 20 mL of DMF, and (8 – *x*) mmol of BzIm and *x* mmol of dcIm were dissolved in 30 mL of DMF. Both solutions were stirred under 393 K for 30 min. After heating, the solutions were cooled down to room temperature. Then, the salt solution was added to the linker mixture and stirred for another 2 h. The powder was collected using a centrifuge with a speed of 14 500 rpm and then washed with fresh DMF three times. For smaller scale preparation, the linker and the salt mixture were stirred at 258 K using an acetone/ice bath by adding acetone to control temperature. In the following membrane preparation, the adsorbed DMF in the framework was exchanged with fresh methanol three times.

### Preparation of Matrimid-Based MMMs

**Scheme 1:**
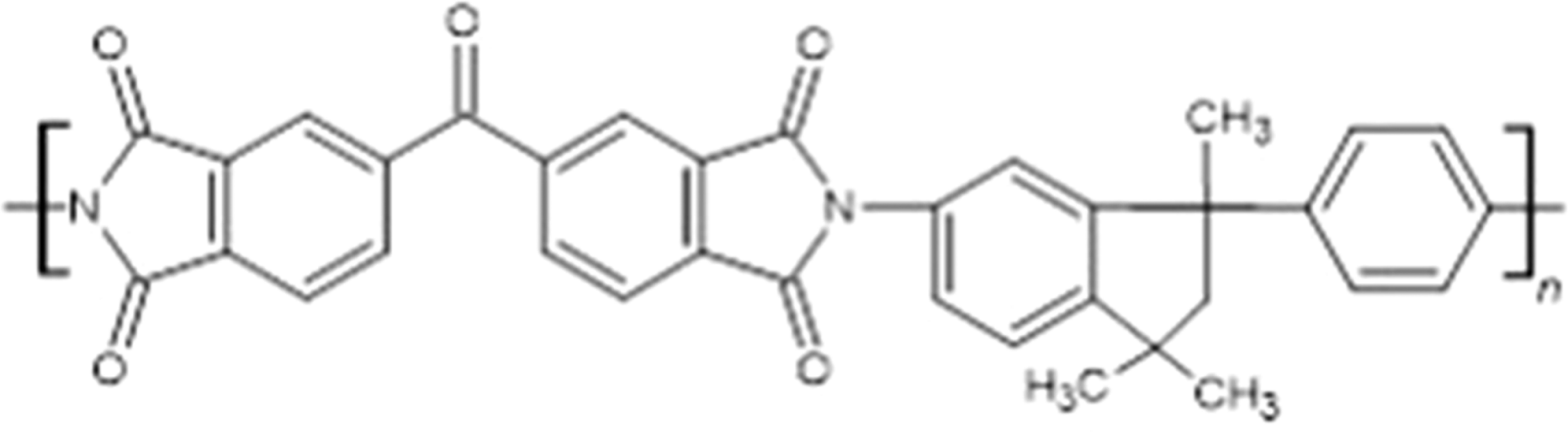
Matrimid Chemical Structure

Matrimid 5218 (Scheme 1) was dried overnight at 100 °C under vacuum. A dried polymer (0.5 g) was dissolved in dichloromethane (CH_2_Cl_2_, 8 g). The solution was stirred for 1 h at room temperature. In the meantime, ZIF crystals [0.03 g (5%), 0.05 g (8%), 0.07 g (12%), 0.12 g (20%)] were suspended in CH_2_Cl_2_ (2 g) by ultrasonication. Then, the two solutions were combined, stirred overnight at ambient temperature, and sonicated for 10 min before casting. The resulting solution was poured into a 5 cm glass Petri dish. The membrane was allowed to form by slow solvent evaporation for 24–36 h in a fume cupboard.

### Preparation of PEBAX-Based MMMs

**Scheme 2:**
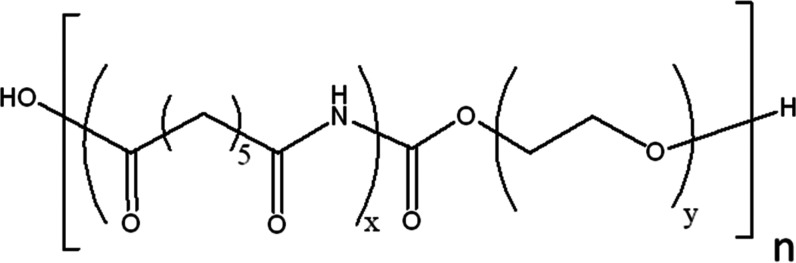
PEBAX MH1657 Chemical Structure (*x* = 40, *y* = 60)

PEBAX MH1657 (Scheme 2, 0.75 g) was dissolved in a water/ethanol mixture (3.5 g/7.9 g) at 353 K under reflux for 3 h. In the meantime, ZIF crystals [0.04 g (5%), 0.1 g (12%), 0.2 g (20%)] were suspended in a water/ethanol mixture (0.5/1 g, 1/2 g, 2/4 g, respectively) by ultrasonication. Then, the two solutions were combined and sonicated for 1 h before casting. The resulting solution was poured onto a glass plate and cast with a doctor blade with a gap of 70 μm. Then, the membrane was covered with a top-drilled box and allowed to dry for 36 h at ambient temperature.

The ZIF content in the above MMMs was calculated by the following equation
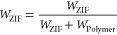
1As a reference, membranes based on neat polymers were also prepared by an identical procedure. The thickness of all membranes was around 30–50 μm, according to averaged measurements performed with a digital micrometer (Mitutoyo) at different locations on each membrane.

### Characterization of ZIF Materials

The crystalline structure of the as-synthesized and dehydrated materials was determined by powder X-ray diffraction (PXRD) on a Stoe STAD I/P diffractometer (Cu K_α1_ X-radiation, λ = 1.54056 Å, 2θ range: 3–40°). Dehydration was performed by loading the sample into 0.7 mm quartz glass capillaries that were then attached to a glass line where they were heated at 473 K for 10 h under a vacuum of 10^–4^ mbar. After dehydration, capillaries were flame-sealed under vacuum. The composition of hybrid ZIFs was analyzed by liquid-state ^1^H nuclear magnetic resonance (NMR) spectroscopy on a Bruker AVII 400, where the relative amounts of the two linkers were quantified after first dissolving the ZIFs. The samples were dissolved by deuterated dimethyl sulfoxide (DMSO) with addition of concentrated HCl (37%) to aid dissolution.^30^ Using the results of these analyses, the samples are described using the nomenclature ZIF-7/COK-17*_xx_*, where *xx* is the percent of the dichloroimidazole linker in the hybrid ZIF (the remainder being benzimidazole).

The morphology of ZIF nanoparticles was examined using electron microscopy (SEM) on a JSM-IT200 instrument. Functional groups were identified by infrared spectroscopy (IR) on a Shimadzu IRAffinity 1S IR spectrometer. Thermogravimetric analysis (TGA) was operated on a Stanton Redcroft STA-780 with a heating rate of 5 K min^–1^. Solid-state NMR spectra were obtained using a Bruker Avance III spectrometer equipped with a 9.4 T wide-bore superconducting magnet (Larmor frequency of 100.6 MHz for ^13^C). Samples were packed into zirconia magic angle spinning (MAS) rotors with outer diameters of 4 mm and rotated at a MAS rate of 12.5 kHz. N_2_ adsorption isotherms were measured volumetrically using a Micromeritics ASAP 2020 gas adsorption analyzer at 77 K. Before measurement, ZIF samples were activated using a tube furnace under a N_2_ atmosphere at 453 K for 10 h. The activated samples were then put under vacuum and heated to the same temperature for 8 h to remove the moisture. High-pressure CO_2_ uptake at 298 K was measured using a Hiden Intelligent Gravimetric Analyzer (IGA). Similar to N_2_ adsorption measurement, the analyzed sample was activated at 453 K for 10 h under vacuum prior to measurement. The mass change was recorded in each adsorption/desorption step, and each step stopped when the uptake reached 98% of the asymptotic equilibrium value or after 90 min, whichever was shorter.

### Crystallography

The structure of the hybrid ZIF, ZIF-7/COK-17_45_ was determined by Rietveld refinement against the PXRD pattern using TOPAS Academic software.^53^ The ZIF-7 framework from the previous literature was selected as the starting model by adapting four distinctive linker placements and *R*3̅ as the space group.^54^ Unlike single-linker ZIF-7, each linker position in this case can be occupied by either BzIm or dcIm, and the occupancies of both linkers on each position were constrained to add to 1. Making use of the fixed geometry of imidazolate linkers, a rigid body strategy was applied to solve the ZIF structure. As such, the groups of atoms connected through chemical bonds within the imidazole linkers were treated as a unit and can be refined simultaneously using the same geometric operation, such as translation and rotation.

### Characterization of MMMs

The crystalline structures of fabricated MMMs were investigated by PXRD in flat plate geometry, on a PANalytical Empyrean diffractometer with Cu K_α1_ radiation and an X’celerator RTMS detector over a 2θ range of 3–40°. The thermal behavior of MMMs was measured by a Netzsch Thermogravimetric Analyzer Jupiter STA449 with a heating rate of 10 K min^–1^. The membranes were examined with a JSM-IT100 (JEOL, Japan) operating at 10 kV. Before SEM analysis, the samples were fractured in liquid nitrogen and then sputtered with a gold layer of 12 nm to form a conductive surface.

### Permeation Measurements

Single gas permeation measurements were carried out using a custom-built constant volume-variable pressure apparatus (Scheme S1) using pure N_2_ and CO_2_ at 1.2 bar and 298 K.

The permeability is obtained from the evolution of the pressure of the downstream side. The permeability coefficient, *P*, was determined from the slope of the pressure versus time curve under the steady-state condition (eq 2)
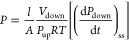
2where *l* is the membrane thickness, *A* is the membrane area, *V*_down_ is the downstream volume, *P*_up_ is the upstream pressure, *P*_down_ is the downstream pressure, *T* is the temperature recorded during analysis, and *R* is the gas constant.

The time lag, θ, was used to determine the diffusivity coefficient *D* (eq 3).
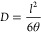
3The solubility coefficient, *S*, for the gas in the polymer was evaluated indirectly, assuming the validity of the diffusion-solution mechanism (eq 4)
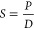
4The ideal selectivity between two gas species *i* and *j* is the ratio of the two single gas permeabilities (eq 5).
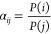
5From the experimental permeation data based on PEBAX_ZIF-7/COK-17_45_ at 12 wt % loading, the intrinsic CO_2_ permeabilities of ZIF-7/COK-17_45_(L) and ZIF-7/COK-17_45_(S) were determined by the Maxwell model (eq 6).
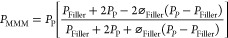
6where *P*_MMM_ is the gas permeability of the mixed matrix membrane, *P*_p_ is the gas permeability of the polymer matrix (PEBAX or Matrimid), *P*_Filler_ is the gas permeability of the filler (ZIF-7/COK-17_45_(L) or ZIF-7/COK-17_45_(S)), and Φ_Filler_ is the volume fraction of the filler inside the MMM.

## Supporting Information Available

Additional PXRD and crystallographic information; SEM micrographs of membranes; TGAs; adsorption isotherms; Maxwell plots; and diagram of apparatus for membrane testing (PDF)
