# Supplementary material for: Hybrid Benzimidazole–Dichloroimidazole Zeolitic Imidazolate Frameworks Based on ZIF-7 and Their Application in Mixed Matrix Membranes for CO2/N2 Separation
Source: ACS Appl Mater Interfaces. 2022 Oct 4;14(41):46615–26. doi: 10.1021/acsami.2c12908 (PMC9585523; doi:10.1021/acsami.2c12908)
Supplement: Supplementary file 1 — am2c12908_si_001.pdf [file am2c12908_si_001.pdf]

Supporting Information

Hybrid benzimidazole-dichloroimidazole zeolitic imidazolate frameworks based on ZIF-7 and their application in mixed matrix membranes for CO_2/N_2 separation

Qian Jia,[a,†] Elsa Lasseuguette,[b,†] Magdalena M. Lozinska,[a] Maria-Chiara Ferrari[b,*] and Paul A. Wright[a,*]

[a] *EaStCHEM School of Chemistry, University of St Andrews, Purdie Building, North Haugh, St Andrews KY16, 9ST, United Kingdom*

[b] *School of Engineering, University of Edinburgh, Robert Stevenson Road, Edinburgh EH9 3FB, United Kingdom*

[†] These authors contributed equally to this work.

Corresponding Authors. M.Ferrari@ed.ac.uk ; paw2@st-andrews.ac.uk

Table S1 Crystal data and structure refinement for ZIF-7/COK-17$_{45}$.

program:	Topas
Crystal system, space group:	Trignoal, R-3
a,c :	22.5502Å, 15.7786Å
α, γ:	90°, 120°
V(Å^3):	6948.641
$R_{wp:}$	6.60%

Site	Type	x	y	z	Occ	Mult
Zn1	Zn	0.6719(1)	0.0705(1)	0.3416(1)	1	18
C1a	C	0.6961(5)	0.0312(6)	0.1798(5)	0.358	18
N2a	N	0.6664(0)	-0.0143(0)	0.1155(3)	0.358	18
N3a	N	0.6505(0)	0.0295(1)	0.2374(8)	0.358	18
C4a	C	0.5990(8)	-0.0461(8)	0.1333(7)	0.358	18
C5a	C	0.5893(1)	-0.0192(8)	0.2082(5)	0.358	18
Cl6a	Cl	0.5375(6)	-0.1107(4)	0.0683(9)	0.358	18
Cl7a	Cl	0.5123(1)	-0.0417(5)	0.2607(1)	0.358	18
CB1a	C	0.6966(6)	0.0301(7)	0.1811(1)	0.642	18
NB2a	N	0.6731(7)	-0.0204(0)	0.1261(1)	0.642	18
NB3a	N	0.6575(4)	0.0229(6)	0.2467(5)	0.642	18
CB4a	C	0.6091(6)	-0.0676(6)	0.1610(3)	0.642	18
CB5a	C	0.6007(1)	-0.0402(5)	0.2342(9)	0.642	18
CB6a	C	0.5396(4)	-0.0777(1)	0.2831(7)	0.642	18
CB7a	C	0.4909(8)	-0.1403(1)	0.2552(8)	0.642	18
CB8a	C	0.4989(1)	-0.1695(4)	0.1793(7)	0.642	18
CB9a	C	0.5580(5)	-0.1340(8)	0.1303(4)	0.642	18
C1b	C	0.7972(4)	0.2009(7)	0.3286(1)	0.779	18
N2b	N	0.8334(6)	0.2560(1)	0.2774(6)	0.779	18
N3b	N	0.7334(5)	0.1567(6)	0.2991(2)	0.779	18
C4b	C	0.7906(4)	0.2461(2)	0.2127(9)	0.779	18
C5b	C	0.7292(3)	0.1852(1)	0.2260(3)	0.779	18
Cl6b	Cl	0.8134(9)	0.3039(4)	0.1281(3)	0.779	18
Cl7b	Cl	0.6559(0)	0.1477(9)	0.1617(3)	0.779	18
CB1b	C	0.7966(9)	0.2018(4)	0.3300(4)	0.221	18
NB2b	N	0.8298(8)	0.2615(3)	0.2917(1)	0.221	18
NB3b	N	0.7309(6)	0.1634(0)	0.3131(0)	0.221	18
CB4b	C	0.7781(1)	0.2621(5)	0.2424(1)	0.221	18
CB5b	C	0.7184(5)	0.2020(4)	0.2564(0)	0.221	18
CB6b	C	0.6570(9)	0.1889(9)	0.2140(1)	0.221	18
CB7b	C	0.6596(3)	0.2372(2)	0.1602(8)	0.221	18
CB8b	C	0.7211(1)	0.2998(9)	0.1450(3)	0.221	18
CB9b	C	0.7818(4)	0.3140(5)	0.1855(7)	0.221	18

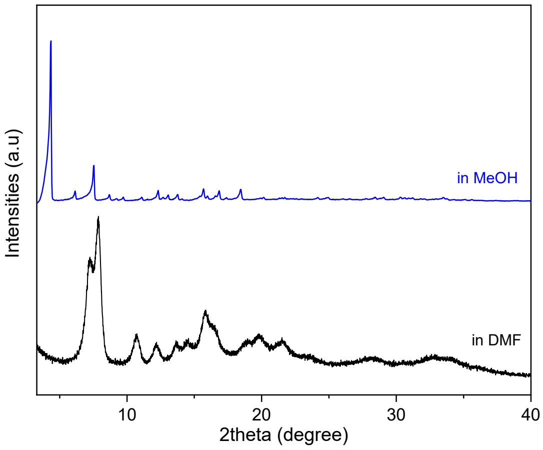

Figure S1 PXRD patterns of hybrid ZIFs with BzIm and dcIm using DMF and methanol synthesis, samples were washed with methanol.

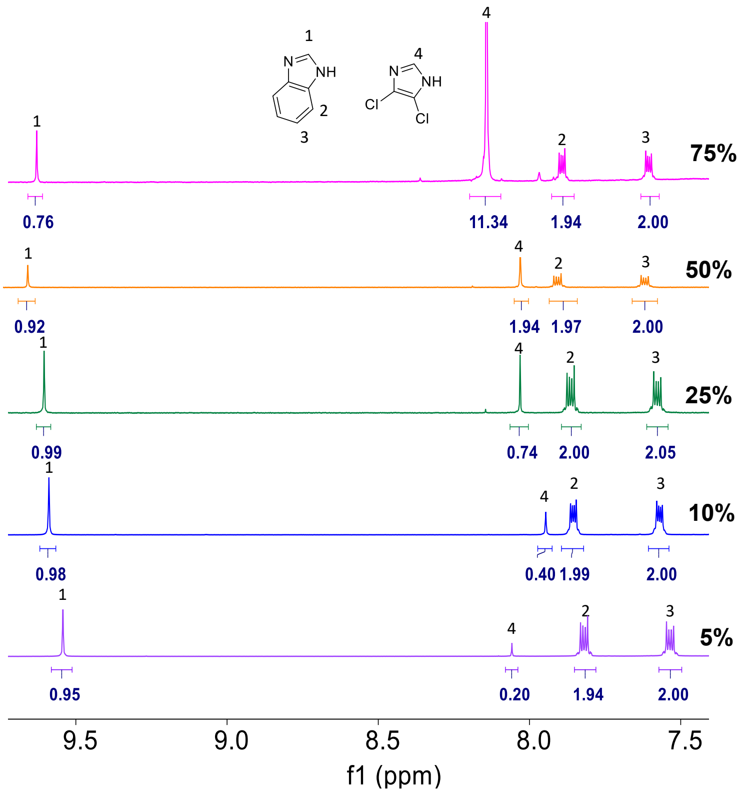

Figure S2 ^{1}H-NMR spectra of hybrid ZIF with BzIm and dcIm. The percentages represent the ratio of dcIm in the synthesis solution.

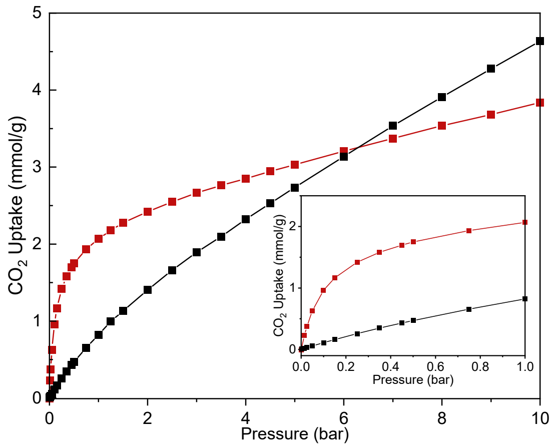

Figure S3 CO_2 adsorption isotherm at 298 K of structures with 45% dcIm: **sod** in red, and **rho** in black.

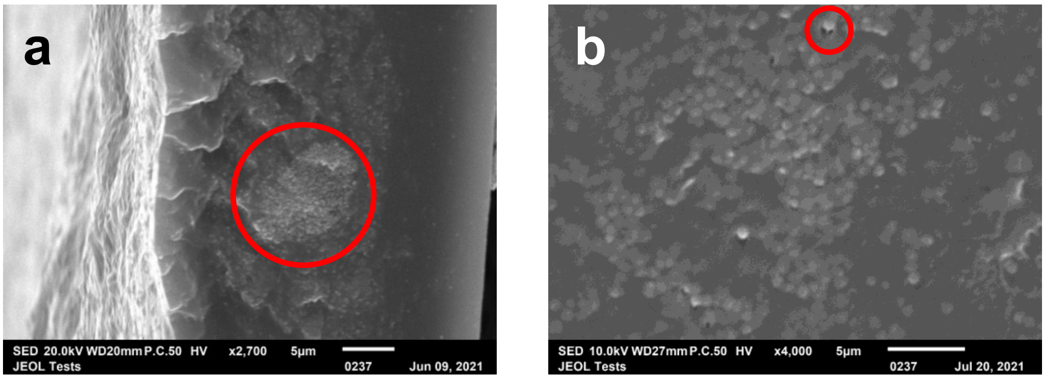

Figure S4 SEM images of (a) membrane cross-section with filler aggolomeration and (b) membrane cross-section with pin-hole.

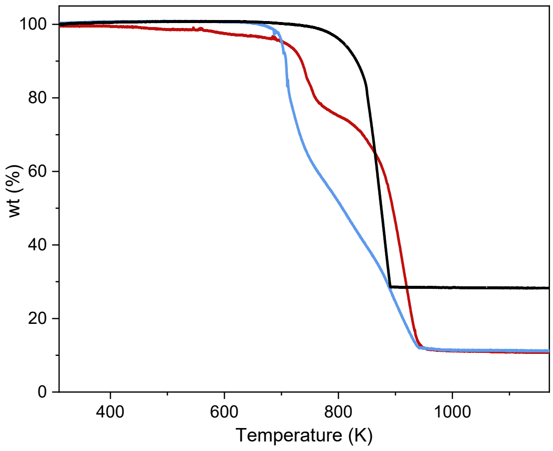

Figure S5 TGA graphs of ZIF-7/COK-17_{45} (in red) compared to ZIF-7 (in black) and COK-17 (in blue).

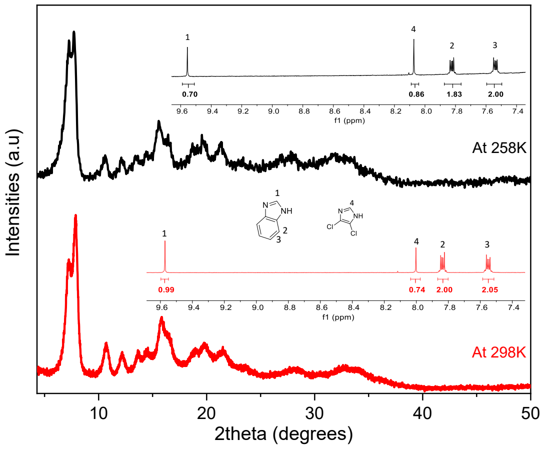

Figure S6 PXRD patterns of ZIF-7/COK-17$_{45}$ synthesized at 258 K and 293 K. Both samples were washed with methanol. Their corresponding solution state ^{1}H NMR spectra are in the insert.

Table S2 Comparison between CO_2 uptake of ZIF-7/COK-17$_{45}$ and ZIF-7-NH_2(70)[1]

Sample	CO_2 uptake [mmol/g]		
	0.1 bar	1 bar	2 bar
ZIF-7/COK-17$_{45}$ (298 K)	0.96	2.06	2.42
ZIF-7-NH_2 (70) (293 K)	0.54	1.65	1.96

Matrimid®

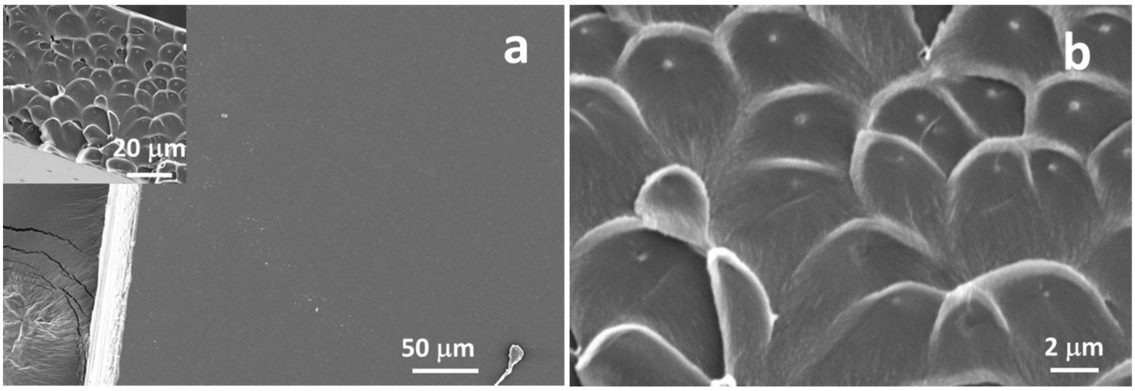

Figure S7 Surface and thickness (a) and cross-sectional (b) SEM images of Matrimid®.

PEBAX

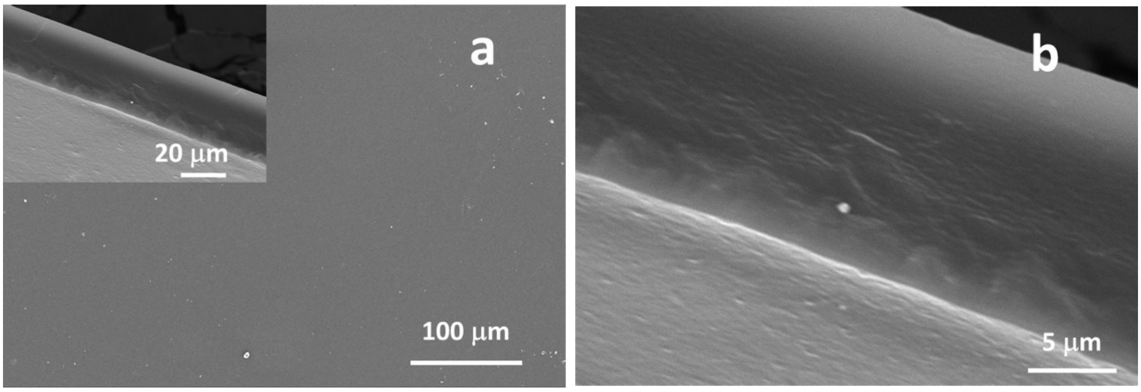

Figure S8 Surface and thickness (a) and cross-sectional (b) SEM images of PEBAX-MH1657.

Matrimid® + ZIF-7/COK-17_{45}(S)

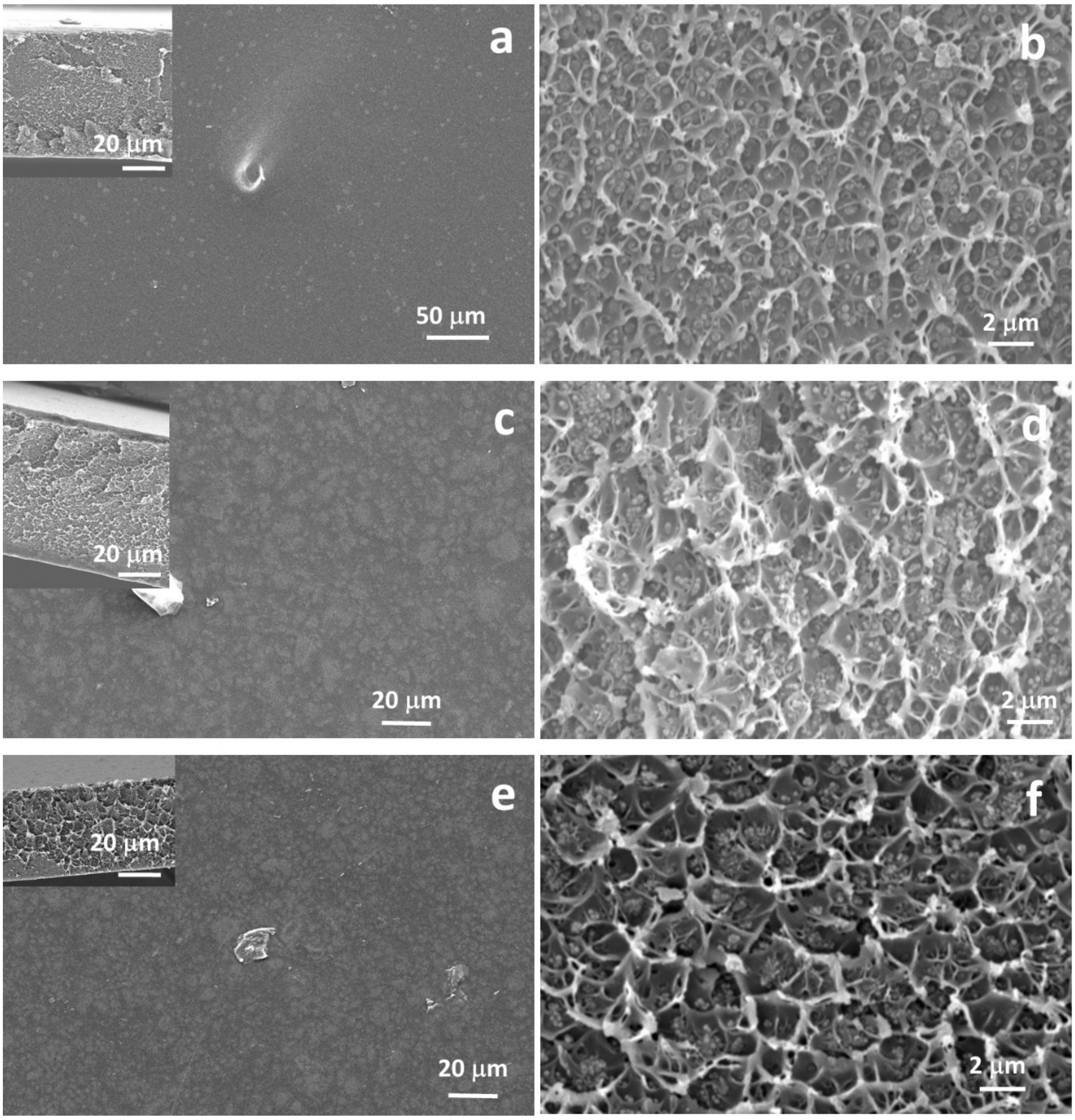

Figure S9 Surface and thickness (a, c, e) and cross-sectional (b, d, f) SEM images of Matrimid®_ ZIF-7/COK-17_{45}(S) with different concentrations of ZIF-7/COK-17_{45}(S): (a, b): 5%, (c, d): 8%, (e, f): 12%.

Matrimid® + ZIF-7/COK-17_{45}(L)

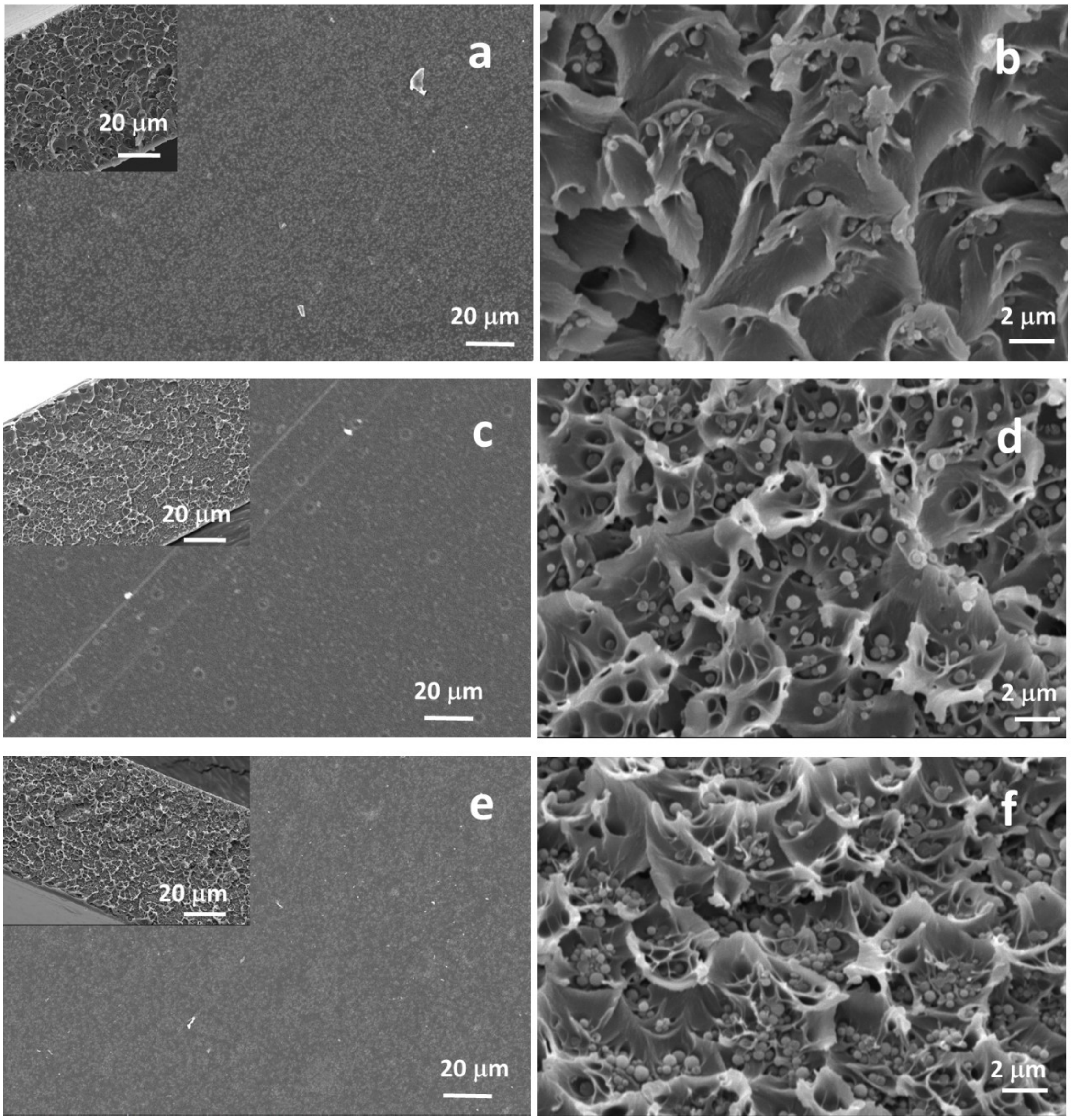

Figure S10 Surface and thickness (a, c, e) and cross-sectional (b, d, f) SEM images of Matrimid®_ ZIF-7/COK-17_{45}(L) with different concentrations of ZIF-7/COK-17_{45}(L): (a, b): 5%, (c, d): 8%, (e, f): 12%.

PEBAX + ZIF-7/COK-17_{45}(S)

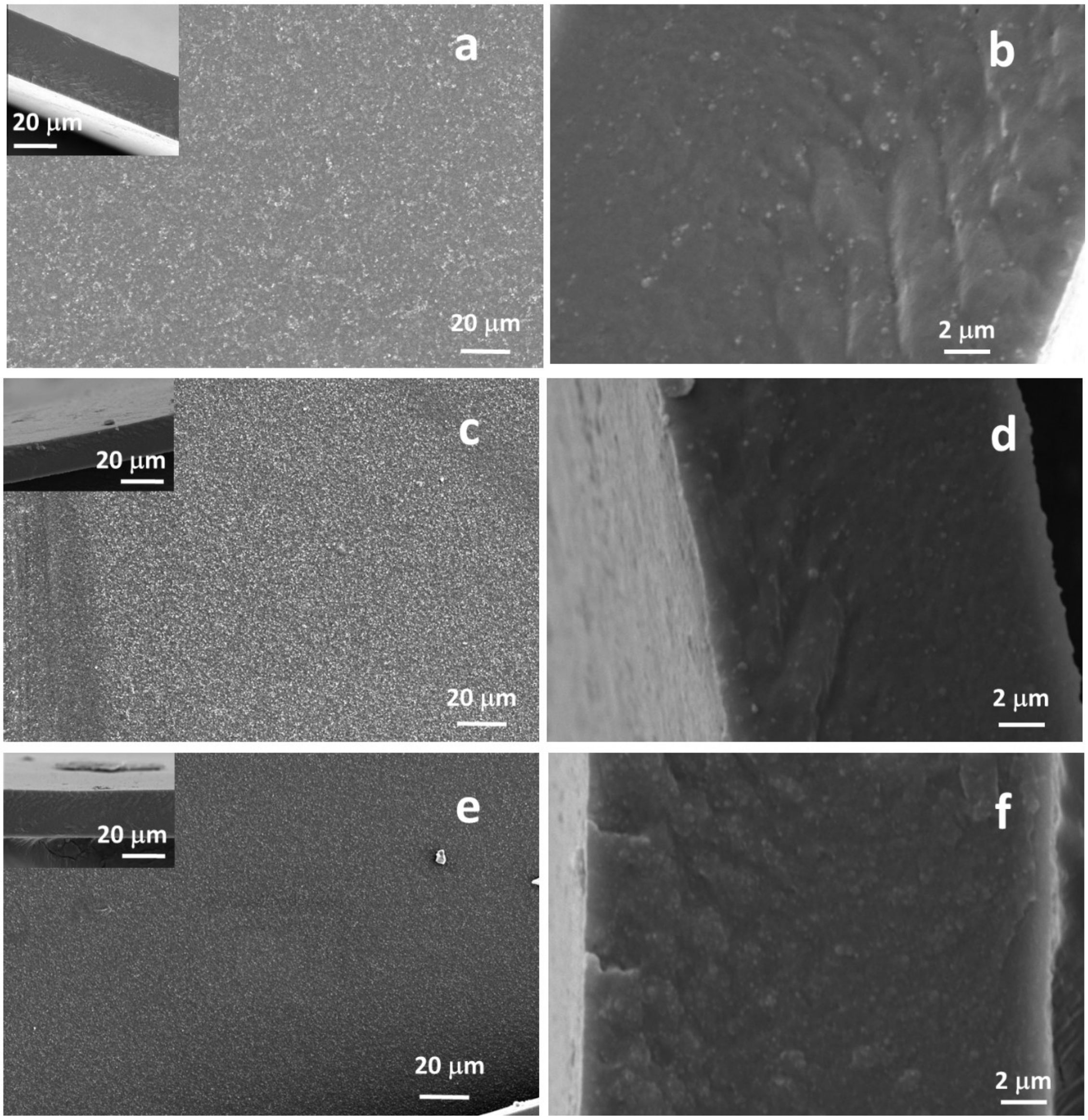

Figure S11 Surface and thickness (a, c, e) and cross-sectional (b, d, f) SEM images of PEBAX_ ZIF-7/COK-17_{45}(S) with different concentrations of ZIF-7/COK-17_{45}(S): (a, b): 5%, (c, d): 12%, (e, f): 20%.

PEBAX + ZIF-7/COK-17_{45}(L)

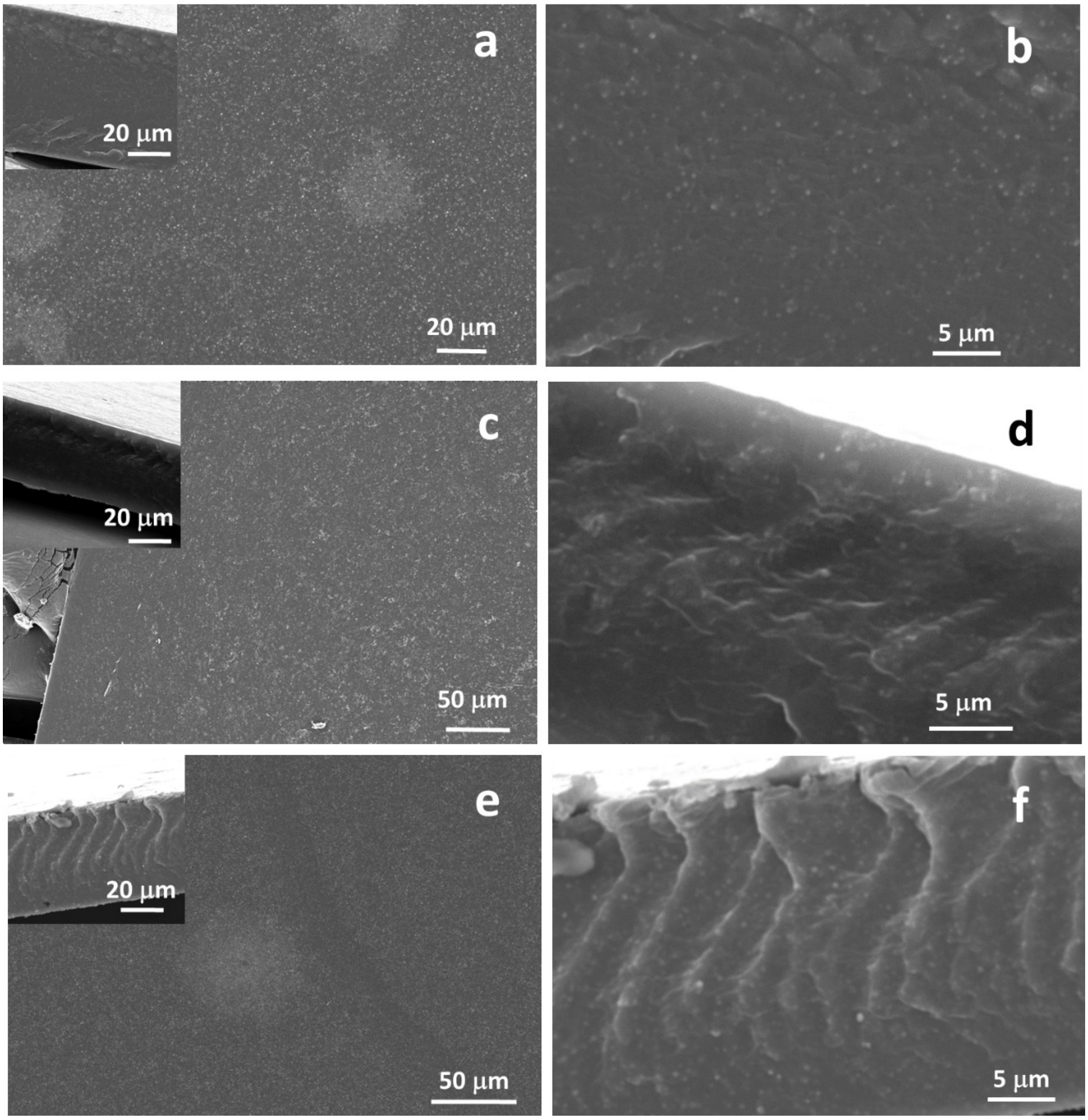

Figure S12 Surface and thickness (a, c, e) and cross-sectional (b, d, f) SEM images of PEBAX_ ZIF-7/COK-17_{45}(L) with different concentrations of ZIF-7/COK-17_{45}(L): (a, b): 5%, (c, d): 12%, (e, f): 20%.

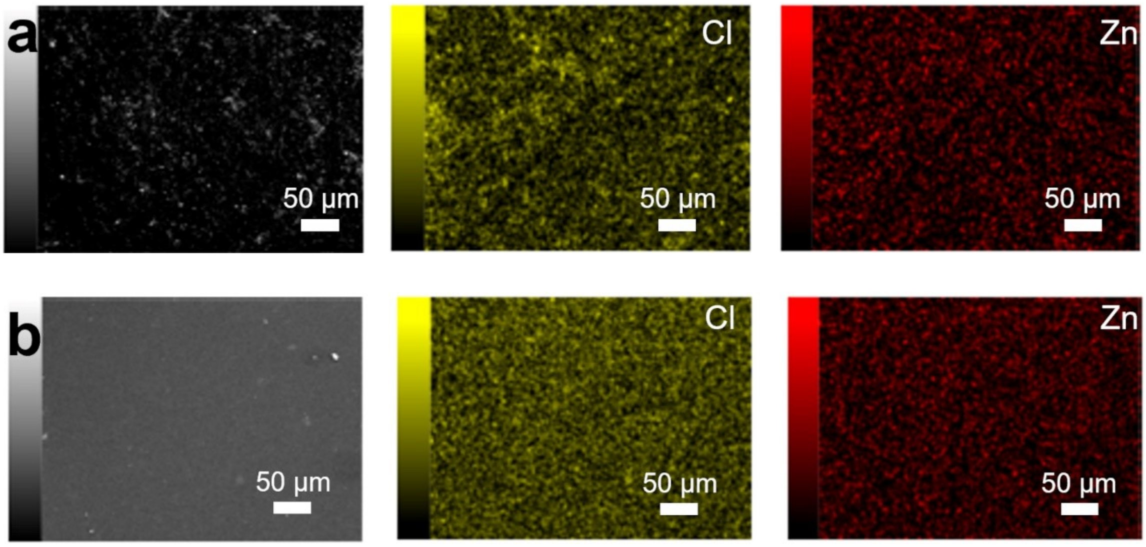

Figure S13 Chlorine and zinc elemental mapping of (a) PEBAX_ZIF-7/COK-17_{45} (L)-12% and (b) Matrimid®_ZIF-7/COK-17_{45} (L)-8%

Table S3 Particle size obtained from SEM images.

Sample	Particle size [nm]
Matrimid®_ ZIF-7/COK-17_{45}(S)-5%	270 *(± 8)*
Matrimid®_ ZIF-7/COK-17_{45}(L)-5%	359 *(± 13)*
PEBAX_ ZIF-7/COK-17_{45}(S)-5%	268 *(± 13)*
PEBAX_ ZIF-7/COK-17_{45}(L)-5%	360 *(± 10)*

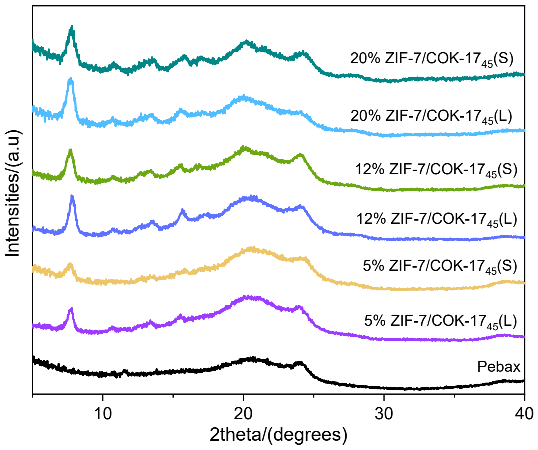

Figure S14 XRD patterns of PEBAX-based MMMs with different fillers and different loading ratio.

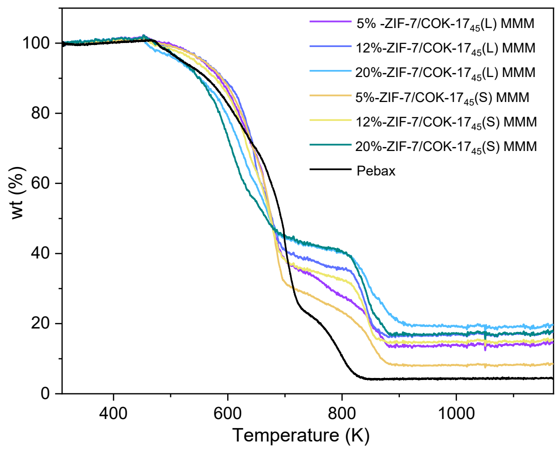

Figure S15 TGA graphs of PEBAX-based MMMs with different fillers and different loading ratio.

Table S4 CO_2 and N_2 permeability, diffusivity and solubility coefficients of pristine Matrimid® and PEBAX, Matrimid_ ZIF-7/COK-17_{45}(S)-5, 8, 12%, Matrimid_ ZIF-7/COK-17_{45}(L)-5, 8, 12%, PEBAX_ ZIF-7/COK-17_{45}(S)-5, 12, 20% and PEBAX_ ZIF-7/COK-17_{45}(L)-5, 12, 20% at 298 K and 1.2 bar

	**Permeability** *[Barrer]*		**Diffusivity** *[10^{-9} $cm^2.s^{-1}$]*		**Solubility** *[10^{-2} $cm^3(STP).cm^{-3}.cmHg^{-1}$]*	
**Sample**	**CO_2**	**N_2**	**CO_2**	**N_2**	**CO_2**	**N_2**
**Matrimid®**	11.4 *(± 1)*	0.34 *(± 0.05)*	22.1 *(± 10)*	15.7 *(± 8)*	13 *(± 8)*	1.2 *(± 0.8)*
**Matrimid_ ZIF-7/COK-17_{45}(S)-5%**	12.7 *(± 3)*	0.35 *(± 0.2)*	6.7 *(± 3)*	-	18.9 *(± 6)*	-
**Matrimid_ ZIF-7/COK-17_{45}(S)-8%**	13.6 *(± 3)*	0.38 *(± 0.2)*	5.9 *(± 3)*	-	22.9 *(± 8)*	-
**Matrimid_ ZIF-7/COK-17_{45}(S)-12%**	15.4 *(± 2)*	0.47 *(± 0.1)*	4.6 *(± 0.6)*	-	33.9 *(± 7)*	-
**Matrimid_ ZIF-7/COK-17_{45}(L)-5%**	14.1 *(± 3)*	0.39 *(± 0.2)*	6.2 *(± 0.7)*	-	22.9 *(± 3)*	-
**Matrimid_ ZIF-7-/COK-17_{45}(L)-8%**	15 *(± 3)*	0.37 *(± 0.2)*	5.8 *(± 0.3)*	-	25.6 *(± 1)*	-
**Matrimid_ ZIF-7/COK-17_{45}(L)-12%**	17.5 *(± 3)*	0.5 *(± 0.2)*	4.7 *(± 0.8)*	-	37.5 *(± 5)*	-
**PEBAX**	49.5 *(± 3)*	1.1 *(± 0.4)*	257 *(± 34)*	158 *(± 100)*	1.9 *(± 0.3)*	0.07 *(± 0.04)*
**PEBAX_ ZIF-7/COK-17_{45}(S)-5%**	62.3 *(± 2)*	0.5 *(± 0.2)*	202 *(± 27)*	136 *(± 34)*	3.1 *(± 0.3)*	0.04 *(± 0.01)*
**PEBAX_ ZIF-7/COK-17_{45}(S)-12%**	64.2 *(± 1)*	0.35 *(± 0.1)*	82 *(± 13)*	40 *(± 3)*	7.8 *(± 0.9)*	0.09 *(± 0.04)*
**PEBAX_ ZIF-7/COK-17_{45}(S)-20%**	39.3 *(± 1)*	0.46 *(± 0.2)*	29 *(± 7)*	47 *(± 4)*	13.4 *(± 2)*	0.1 *(± 0.07)*
**PEBAX_ ZIF-7/COK-17_{45}(L)-5%**	61.4 *(± 2)*	1.1 *(± 0.4)*	160 *(± 13)*	191 *(± 100)*	3.8 *(± 0.3)*	0.05 *(± 0.04)*
**PEBAX_ ZIF-7/COK-17_{45}(L)-12%**	68 *(± 1)*	0.86 *(± 0.1)*	97 *(± 4)*	103 *(± 24)*	7 *(± 1)*	0.09 *(± 0.02)*
**PEBAX_ ZIF-7/COK-17_{45}(L)-20%**	42 *(± 1)*	0.83 *(± 0.2)*	42 *(± 3)*	41 *(± 20)*	10 *(± 0.7)*	0.2 *(± 0.1)*

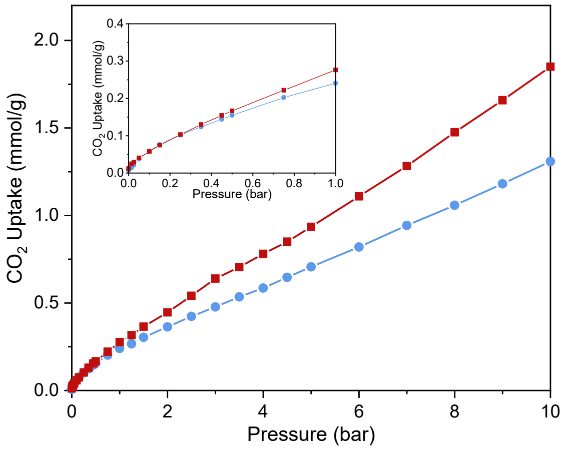

Figure S16 CO_2 uptake at 298 K of PEBAX-based MMMs with ZIF-7/COK-17_{45}(S) (in red) and ZIF-7/COK-17_{45}(L) (in blue) at the loading of 12%.

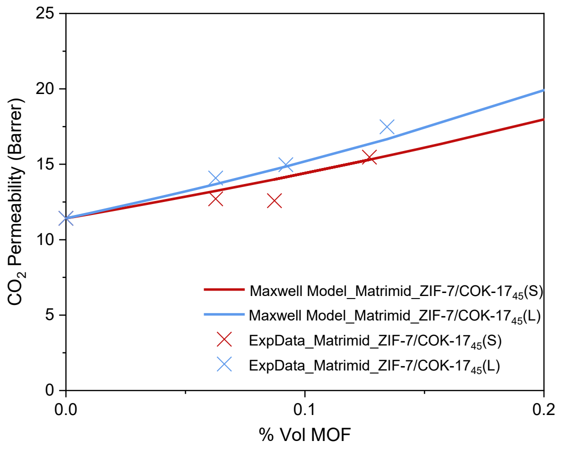

Figure S17 Experimental (Cross) and theoretical (Line) CO_2 permeability for MMM based on Matrimid® and ZIF-7/COK-17_{45} as a function of the % vol of MOF (Blue: ZIF-7/COK-17_{45}(L) and Orange: (ZIF-7/COK-17_{45}(S)).

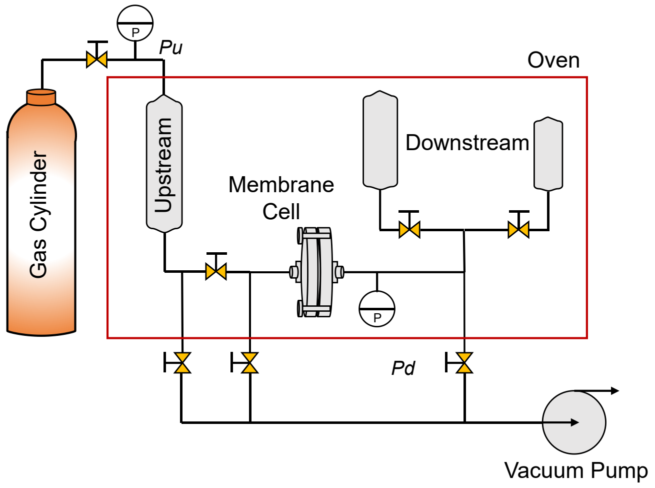

Scheme S1 Constant volume-variable pressure apparatus.

References

1. Xiang, L. *et al.* Amino-Functionalized ZIF-7 Nanocrystals: Improved Intrinsic Separation Ability and Interfacial Compatibility in Mixed-Matrix Membranes for CO_2/CH_4 Separation. *Adv. Mater.* **2017**, *29*, 1606999.
